# UVA-Degradable Collagenase Nanocapsules as a Potential Treatment for Fibrotic Diseases

**DOI:** 10.3390/pharmaceutics13040499

**Published:** 2021-04-06

**Authors:** Víctor M. Moreno, Carolina Meroño, Alejandro Baeza, Alicia Usategui, Pablo L. Ortiz-Romero, José L. Pablos, María Vallet-Regí

**Affiliations:** 1 Departamento de Química en Ciencias Farmacéuticas, Universidad Complutense de Madrid, Instituto de Investigación Sanitaria, Hospital 12 de Octubre i+12, Plaza Ramón y Cajal s/n, 28040 Madrid, Spain; victomor@ucm.es; 2 CIBER de Bioingeniería, Biomateriales y Nanomedicina, CIBER-BBN, 28029 Madrid, Spain; 3 Servicio de Reumatología, Instituto de Investigación Hospital 12 de Octubre (i+12), Universidad Complutense de Madrid, Avenida Córdoba s/n, 28041 Madrid, Spain; camerono@ucm.es (C.M.); ausategui.imas12@h12o.es (A.U.); jlpablos@h12o.es (J.L.P.); 4 Departamento de Materiales y Producción Aeroespacial, ETSI Aeronáutica y del Espacio, Universidad Politécnica de Madrid, 28040 Madrid, Spain; 5 Servicio de Dermatología, Hospital 12 de Octubre, Instituto (i+12 Medical School), Universidad Complutense de Madrid, Avenida Córdoba s/n, 28041 Madrid, Spain; pablo.ortiz@salud.madrid.org

**Keywords:** photolinker, collagenase nanocapsules, fibrotic diseases, collagen, UVA

## Abstract

Peyronie and Dupuytren are pathologies characterized by the appearance of localized fibrotic lesions in an organ. These disorders originate from an excessive production of collagen in the tissue provoking dysfunction and functional limitations to the patients. Local administration of collagenase is the most used treatment for these fibrotic-type diseases, but a high lability of the enzyme limits its therapeutic efficacy. Herein, we present a novel methodology for the preparation of collagenase nanocapsules without affecting its enzymatic activity and capable of releasing the enzyme in response to an ultraviolet A (UVA) light stimulus. Polymeric coating around collagenase was formed by free-radical polymerization of acrylamide-type monomers. Their degradation capacity under UVA irradiation was provided by incorporating a novel photocleavable acrylamide-type crosslinker within the polymeric framework. This property allowed collagenase release to be triggered in a controlled manner by employing an easily focused stimulus. Additionally, UVA irradiation presents considerable benefits by itself due to its capacity to induce collagenase production in situ. An expected synergistic effect of collagenase nanocapsules in conjunction with UVA effect may present a promising treatment for these fibrotic diseases.

## 1. Introduction

Fibrosis is defined as the excessive overproduction and accumulation of collagens and other extracellular matrix (ECM) proteins in the connective tissue of different organs. This pathology usually leads to discomfort and malfunction or disablement of the affected organ [1,2]. Collagen is synthesized by fibroblasts and other mesenchymal cells, such as myofibroblasts. These cells are ultimately responsible for the overproduction of ECM-related proteins. This overproduction leads, in turn, to the accumulation of excessive amounts of collagens, and the contraction and remodeling of the connective tissue [3]. The inappropriate remodeling of ECM lead to the formation of fibrotic lesions fibrosis, which are characterized by scarring and thickening of the affected tissue [4,5]. The process may occur systemically (i.e., systemic sclerosis [6]) or locally (i.e., liver cirrhosis [7]) as an abnormal repairing process following inflammatory lesions. This can also be observed in pathological wound healing, leading to abnormal, hypertrophic or keloid scars, as consequence of autoimmune conditions. This process is observable in different fibrotic-type diseases, such as scleroderma (morphea) [8], and those with localized fibrotic lesions that frequently cause severe local dysfunction (i.e., painful erections in Peyronie disease [9,10] or hand contractures in Dupuytren disease [11,12]). These pathologies are characterized by an overproduction of collagen, which induces local discomfort and functional limitation to the patients.

These diseases are mainly treated by surgery or collagenase administration [13]. Surgical procedures are invasive interventions that frequently involve long recovery times for patients. Restoration of a proper functionality is challenging, and relapses are not rare [11]. Furthermore, several side effects as tendon, nerve, or artery injuries are commonly found. Since Food and Drug Administration (FDA) approved in 2010 the injection of Collagenase Clostridium Histolyticum (CCH) for the intra-lesional treatment of Peyronie’s disease and Dupuytren’s contracture [14], collagenase has become the preferred treatment for these localized pathologies. CCH is a matrix metalloproteinase that specifically cleaves type I and III fibrillar collagens without affecting type IV collagen. Type I and III are the major collagen fibers in ECM, while type IV are mainly present in basement membranes [3]. Since fibrotic lesions are produced in ECM, local administration of CCH promote a meaningful reduction of the excessive collagen fibers present in fibrotic tissues, alleviating thereby the painful symptoms of these diseases [13,15]. The main limitation of the use of this enzyme is its high liability in physiological environment; collagenase loses its activity in less than 24 h at physiological conditions. This issue may be solved by periodically injecting multiple collagenase doses but produces important local pain and is time consuming for patients and health professionals. On the other hand, there is a potential risk of adverse events, derived from overdose, leading to tissue damage.

Our research group has developed diverse methodologies to produce polymeric nanocapsules for a wide variety of enzymes; as peroxidase [16], catalase [17] or collagenase [18]. Encapsulation protects the enzyme from degradation by external factors, but do not affect its enzymatic activity, which are of interest in the delivery of therapeutic enzymes in various diseases. In this way, collagenase nanocapsules were designed to release enzymes in a controlled manner into the diseased tissue in response to a local stimulus, such as pH changes. This feature has been employed to degrade the ECM in tumoral-like tissues, which exhibit acidic environment. The pH-promoted collagenase release enhanced the penetration of a drug-loaded nanocarrier [19]. Latterly, the incorporation of a non-degradable crosslinker within nanocapsules structure reduced the hydrolysis rate. In this way, the release of controlled amounts of collagenase was achieved over more than 10 days, which was advantageous for the treatment of skin fibrosis [20]. Additionally, the dual-fluorescent labelling of nanocapsules allowed the monitorization of their penetration in a three-dimensional collagen gel [21].

Herein, we present a novel strategy for the controlled release of collagenase using light as stimulus. This fact was achieved by incorporating a novel light-sensitive crosslinker monomer in the polymeric framework of synthetized collagenase nanocapsules. Photosensitive compounds have been used as cleavable linkers in a broad variety of biomedical applications such as drug or biomolecule conjugates [22], disassembly of supramolecular structures [23], or photo-controlled release of therapeutics from drug delivery systems [24,25]. In this case, UVA-light sensitivity was provided by the photolabile *α*-methyl-*o*-nitroveratryl (ONV) moiety present in the photosensitive crosslinker [26,27,28,29,30]. This property triggers the collagenase release in specific regions employing an easy-to-focus stimulus. Through this external control, the administered dosage of collagenase is more controllable, avoiding overdoses if the free collagenase is administered. Moreover, it has been established that UVA phototherapy may reduce skin thickness and stiffness [31]. UVA irradiation may provide a benefit in the treatment of fibrotic lesions due to its potential anti-inflammatory effect and its ability to stimulate the production of collagenase in situ [32,33]. Therefore, a synergistic effect of UVA with collagenase nanocapsules might improve their therapeutic effect.

## 2. Experimental Section

### 2.1. Materials

All chemicals and reagents were bought from the corresponding supplier and used without further purification. Sodium Phosphate tribasic pure anhydrous (Acros Organics, Waltham, MA, USA); Collagenase Type I (Life Technologies, Waltham, MA, USA); Acrylamide (Fluka, Madrid, Spain); 2-Aminoethyl methacrylate hydrochloride (Sigma Aldrich, Madrid, Spain); Ammonium persulfate (APS) (Sigma Aldrich, Madrid, Spain); N,N,N’,N’-Tetramethylethylenediamine (TMEDA) (Sigma Aldrich, Madrid, Spain); Amicon^®^ Ultra-2 mL Centrifugal Filters Ultracel^®^-10K (Millipore, Darmstadt, Germany); Ethanol absolute 99.5% for Synthesis (Panreac, Barcelone, Spain); Mono-Fmoc-diethylamine (Iris Biotech, Marktredwitz, Germany); 25% Ammonia aqueous solution, Dichloromethane (DCM), Methanol (MeOH), Sodium hydroxide (NaOH), Dimethyl sulfoxide (DMSO), DMSO-d_6_, ISOLUTE^®^ SCX-2 cartridge, Doxorubicin hydrochloride (Dox), N-diisopropyethylamine (DIPEA), Piperidine, anhydrous N,N-dimethylformamide (DMF) and Acryloyl chloride were purchased from Sigma-Aldrich. N,N,N′,N-Tetramethyl-O-(1H-benzotriazol-1-yl)-uronium hexafluorophosphate (HBTU), 1-Hydroxybenzotriazole (HOBt) and 4-{4-[1-(9-Fluorenylmethyloxycarbonylamino)ethyl]-2-methoxy-5-nitrophenoxy}butanoic acid (Fmoc-Photolinker) were purchased from Abcr (Karlsruhe, Germany)

EnChek^®^Gelatinase/Collagenase Assay Kit (Life Technologies, Waltham, MA, USA); Soluble Collagen Quantification Assay Kit (Sigma-Aldrich, Madrid, Spain); Cell Proliferation WST-1 Assay (Roche Applied Science, Rotkreuz, Switzerland); Dulbecco′s Modified Eagle′s Medium (DMEM) (Lonza, Verviers, Belgium); Fetal bovine serum (FBS) (HyClone, Cramlington, UK); L-Glutamine, trypsin-EDTA and Collagen Type I, Rat (Gibco, Paisley, UK); 1% penicillin/streptomycin (Lonza, Waltham, MA, USA).

Characterization: The photolinker was irradiated using a Fischer Scientific UVP Blak-Ray™ B-100AP High-Intensity UV Lamp (100 W, 230 V, 50 Hz, wavelength 365 nm). Nanocapsules were irradiated with a Medisun HF-54 UVA-phototherapy device. The hydrodynamic size of protein capsules was measured by means of a Zetasizer Nano ZS (Malvern Instruments) equipped with a 633 nm “red” laser. Dinamic Light Scattering (DLS) was carried out using a Malvern Zetasizer Nano. Transmission Electron Microscopy (TEM) was carried out with a JEOL JEM 3000 instruments operated at 300 kV, equipped with a CCD camera. Sample preparation was performed by dispersing in distilled water and subsequent deposition onto carbon coated copper grids. A solution of 1% of phosphotungstic acid (PTA) pH 7.0 was employed as a staining agent, in order to visualize the protein capsules. Fourier Transform infrared (FTIR) spectroscopy was performed with a Thermo Nicolet nexus equipped with a Goldengate attenuated total reflectance device (ATR-FTIR) for the identification of functional organic groups. ^1^H Nuclear Magnetic Resonance (NMR), ^13^C-NMR, COSY 2D-NMR and HMQC 2D-NMR spectra were recorded on a Bruker AV (250 MHz) spectrometer at room temperature. Chemical shifts were recorded in parts per million (ppm). Matrix-Assisted Laser Desorption/Ionization—Time-Of-Flight (Maldi-TOF-TOF) and Electrospray ionization mass spectra for the synthesized molecules (Gathered at Supporting Information) were obtained at Electron Microscopy Centre, UCM. LyoQuest laboratory freeze dryer was employed for lyophilization.

### 2.2. Bisacrylamide-Photolinker (PL) Synthesis and Photosensitivity Evaluation

Fmoc-PL-NHFmoc (1) synthesis. To a solution of 4-{4-[1-(9-Fluorenylmethyloxycarbonylamino)ethyl]-2-methoxy-5-nitrophenoxy}butanoic acid (Fmoc-Photolinker) (500 mg, 0.96 mmol), HOBt (1.91 mmol) and HBTU (1.91 mmol) in 8 mL of anhydrous DMF, DIPEA (5.25 mmol) was slowly added. The solution was cooled on ice and a solution of mono-Fmoc-ethylene diamine (1.43 mmol) in 3 mL of anhydrous DMF, also cooled on ice, was added dropwise. After stirring for 30 min at 0 °C, the mixture was stirred overnight at room temperature under inert atmosphere. The resulting precipitate was filtered and washed with DMF followed by in vacuo drying. Product **1** (average yield ~68%) was obtained as an amorphous white powder. ^1^H NMR (250 MHz, DMSO-d_6_) δ 7.89 (d, 4H), 7.67 (t, 4H), 7.48 (s, 1H), 7.45–7.26 (m, 8H), 7.25 (s, 1H), 5.19 (t, 1H), 4.34–4.25 (m, 4H), 4.26–4.13 (m, 2H), 4.03 (t, 2H), 3.86 (s, 3H), 3.14–3.01 (m, 4H), 2.22 (t, 2H), 2.01–1.87 (m, 2H), 1.42 (d, 3H).

Diamine-Photolinker (2) synthesis. A quantity of 4 mL of Piperidine were added dropwise to a solution of **1** (0.65 mmol) in anhydrous DMF (16 mL) until reach a final concentration of 20% Piperidine in DMF. The solution was stirred at room temperature for 18 h, and then DMF was removed by evaporation. The obtained crude was dissolved in cold MeOH, and the resulting precipitate was removed by filtration. After evaporating the filtrated solution, product was purified by Strong Cation Exchange (SCX) using a solution of Ammonia in MeOH as eluent. Product 2 was obtained as a pale-yellow liquid with a yield of 42%. ^1^H NMR (250 MHz, MeOD) δ 7.53 (s, 1H), 7.33 (s, 1H), 4.64 (q, 1H), 4.10 (t, 2H), 3.99 (s, 3H), 3.29 (t, 2H), 2.76 (t, 2H), 2.45 (t, 2H), 2.19–2.07 (m, 2H), 1.46 (d, 3H).

Bisacrylamide-Photolinker (3) synthesis. To a solution of 2 (0.273 mmol) in 15 mL of anhydrous DMF at 0 °C was added dropwise DIPEA (1.09 mmol) and acryloyl chloride (0.95 mmol) under agitation. Reaction mixture was stirred overnight at room temperature. Next day, solution was evaporated under vacuum and the resulting crude was redissolved in DCM. The organic layer was washed (x3) with 5% LiCl (aq), brine and water. Then was dried with sodium sulphate and evaporated to afford the product **3** as a yellow solid with a yield of 55%. ^1^H NMR (250 MHz, MeOD) δ 7.59 (s, 1H), 7.08 (s, 1H), 6.40–6.14 (m, 4H), 5.67 (dd, 2H), 5.66 (t, 1H), 4.09 (t, 2H), 3.93 (s, 3H), 3.37 (t, 2H), 3.36 (t, 2H), 2.42 (t, 2H), 2.15–2.05 (m, 2H), 1.57 (d, 3H). ^13^C NMR (63 MHz, DMSO-d_6_) δ 172.55, 165.64, 164.73, 154.21, 147.18, 140.90, 135.91, 132.64, 132.34, 126.49, 125.88, 110.74, 108.96, 69.12, 57.26, 44.76, 39.31, 39.19, 32.48, 25.46, 22.69.

Photolysis assay. 2.5 mg of the synthesized product 3 were diluted in a volume of 1 mL of DMSO-d_6_ and transferred to a 1 × 1 cm quartz translucent cuvette. Then, the solution was exposed to three cycles of irradiation (10 min of UVA exposure each) using an irradiation wavelength of 365 nm at a distance of ∼20 cm from the lamp focus using a Fischer Scientific UVP Blak-Ray™ B-100AP High-Intensity UV Lamp (100 W, 230 V, 50 Hz, wavelength 365 nm). Resulting solution for each irradiation time was analyzed by ^1^H NMR.

### 2.3. Synthesis of UVA-Degradable Collagenase Nanocapsules (nCol-PL)

This methodology is a modification from our previously reported works for the obtention of collagenase nanocapsules [18,20,21]. Firstly, a 0.01 M NaHCO_3_ buffer solution at pH 8.5 was fully deoxygenated by three freeze-vacuum cycles under N_2_ atmosphere. Then, 0.026 mmol of 2-aminoethylmetacrylate hydrochloride (AEM) and 0.035 mmol of acrylamide (Am) dissolved in 1 mL of deoxygenated buffer were added to a 1 mL solution of type I collagenase (3.1 · 10^−5^ mmol) in the same buffer. Subsequently, 0.01 mmol of bisacrylamide-Photolinker (product 3) dissolved in 50 µL of DMSO were added dropwise to this mixture under agitation. The resulting mixture was kept at 300 rpm for 10 min under orbitalic agitation at N_2_ atmosphere and room temperature. Next, radical initiators TMEDA (0.02 mmol) and APS (0.013 mmol) dissolved in 1 mL of deoxygenated buffer were added to the mixture. The reaction mixture was then kept at 300 rpm for 90 min under orbitalic agitation at N_2_ atmosphere and room temperature. Finally, the obtained nanocapsules were centrifugated in 10 kDa cut-off filters (AMICON) and washed thrice with the NaHCO_3_ buffer to remove the unreacted monomers. Resulting UVA-degradable collagenase nanocapsules (*nCol-PL*) were diluted to a 1 mL volume and were preserved from light at 4 °C. Determination of protein concentration was carried out by colorimetric Bicinchoninic acid (BCA) Assay [34]. The protocol consisted on the addition of 50 µL of the protein sample to 200 µL of a “working solution” composed by Copper (II) Sulfate and Bicinchoninic acid. This mixture was incubated for 30 min at 40 °C and then, absorbance at 562 nm was measured.

### 2.4. Nanocapsules Photocleavage Evaluation

Synthetized nCol-PL were diluted to 50 µg/mL with a 0.01 M NaHCO_3_ buffer solution at pH 8.5, and this solution was placed into a 1 × 1 cm quartz translucent cuvette. Subsequently, cuvette was irradiated with UVA light (λ = 365 nm) for 10 min, at a distance of 20 cm between cuvette and the lightbulb. The solution was irradiated using a High-Intensity UV Lamp (UVP Blak-Ray™ B-100AP from Fischer Scientific). After 1 h, the irradiated solution of nanocapsules was analyzed by DLS and TEM.

### 2.5. Nanocapsules Enzymatic Activity Measurements

The enzymatic activity of free collagenase and nCol-PL was evaluated using and following the protocol EnzChek Gelatinase/Collagenase Assay Kit. For this experiment, 80 μL of phosphate buffer 1×, 20 μL of Collagen-FITC, and 100 μL of each collagenase sample were used. All samples were standardized to an enzymatic concentration of 0.2 U/mL in 1× Phosphate buffered saline (PBS) solution at pH 7 at room temperature and their enzymatic activity was studied at different times measuring the fluorescence intensity in a fluorescence microplate reader (F_abs_ = 495 nm/F_em_ = 515 nm).

### 2.6. Nanocapsules Collagen Gel Digestion Capacity

nCol-PL kinetics of collagen digestion was evaluated using a synthetic three-dimensional (3D) collagen gel as a substrate and using the Soluble Collagen Quantification Assay Kit (Fluorometric assay).

Three-dimensional (3D) collagen gel preparation: Briefly, 2 mL of Rat Tail Collagen type I (3 mg·ml^–1^) and 0.6 mL of complemented DMEM (DMEM medium with 10% FBS and l-Glutamine) were mixed at 0 °C and subsequently 100 µL of a 2 M NaOH solution was added until neutral pH. Then, 0.5 mL of FBS and 1 mL of 1× PBS were added to the solution at 0 °C. Afterwards, 50 µL of this mixture was added to the wells of a 96-well plate and incubated at 37 °C at an atmosphere of 5% CO_2_ for 24 h to promote the collagen gelification. The resulting collagen gels (73 µg of collagen per well) were employed for the further experiment one day after gels formation.

Collagen degradation kinetics: nCol-PL were diluted in PBS to a final concentration of 20 µg/mL and 80 µL were added to each well containing collagen gel. Then, nCol-PL were activated by exposure to UVA light with a Medisun HF-54 UVA-phototherapy device, irradiating placed 10 cm above from the plates, and kept for 15 min, reaching a final UVA dosage of 30 J/cm^2^. Straightaway, the plates were incubated (37 °C, 5% CO_2_) for different times: 0 h, 30 min, 1 h, 2 h, 4 h, 6 h, 8 h and 24 h and supernatants were collected and immediately frozen at −80 °C. A control for each analysis time was also prepared with the addition of PBS on the collagen gel. Collagen degradation was measured by the quantification of its degradation products using the Soluble Collagen Quantification Assay Kit (Fluorometric) following manufacturer’s protocol. The absorbance was measured at 465 nm.

### 2.7. In Vitro Cytotoxicity Evaluation of nCol-PL

Cell culture: Primary Skin fibroblasts from either healthy individuals or from patients suffering cutaneous scleroderma were cultured in DMEM supplemented with 10% FBS, 1% glutamine and 1% Penicillin/Streptomycin solution. Cultured cells were maintained at 37 °C in 5% CO_2_ and medium was replaced every 3 days. Cells were passaged when 90% confluence was reached using trypsin-EDTA.

Cell viability assay: nCol-PL citotoxicity was tested in vitro using cultured SK-FB from both healthy individuals and from patients suffering cutaneous scleroderma. Cells were seeded in 96-well plates (5 · 10^3^ cells/well) and incubated overnight at 37 °C and 5% CO_2_). Then, cells were treated with different nCol-PL concentrations and exposed to 30 J/cm^2^ UVA irradiation or remained untreated. nCol-PL solutions were prepared in a concentration range of 6.25 to 500 µg/mL in the cell culture medium and were added in a final volume of 100 µL per well. Six replicates were carried out for each sample. After this, cells were incubated for 24 h (37 °C, 5% CO_2_), and on the next day, the cell viability assay was performed with Cell Proliferation WST-1 Assay, following manufacturer’s protocol. For this, cell viability was quantified by measuring absorbance after 2 h at 450 nm for the sample and at 650 nm for the background. Blank was assessed as medium alone with WST-1 and was subtracted from all values. Values of WST-1 were normalized to the untreated control and represented as fold-change values.

## 3. Results and Discussion

In this work, our strategy is based on the encapsulation of the proteolytic enzyme collagenase into a tiny nanometric polymeric capsule, which is sensitive to UVA radiation in such a way that this capsule can be immediately disassembled by the UVA radiation exposition. This novel strategy involves the synthesis of UVA-sensitive crosslinkers, which will be incorporated in the composition of the polymeric nanocapsules to provide light-induced release behavior. The efficient formation of the polymeric capsule around the protein requires a balanced interaction of the monomers, and crosslinkers with the protein surface, which are ruled by intermolecular forces (hydrogen bonds, Van der Waals, etc.). The different chemical nature of the UVA-sensitive crosslinker, in comparison with the degradable ones, previously employed in the group, complicates the formation of the capsules around the enzyme. This leads to poor encapsulation yield or incomplete coating of the enzymes. Therefore, in order to overcome this problem, the polymerization process was evaluated using different monomers and ratios so that the best conditions were identified for efficient encapsulation and UVA-sensitive behavior.

### 3.1. Synthesis of UVA Sensitive Photolinker

In order to correctly incorporate ONV moiety in the structure of nanocapsules, acrylate groups in both ends of the molecule were required. The presence of an α,β-unsaturated carbonyl in the photolabile compound provides the capacity to react intermolecularly between monomers and crosslinkers during the free-radical polymerization reaction.

**Scheme 1:**
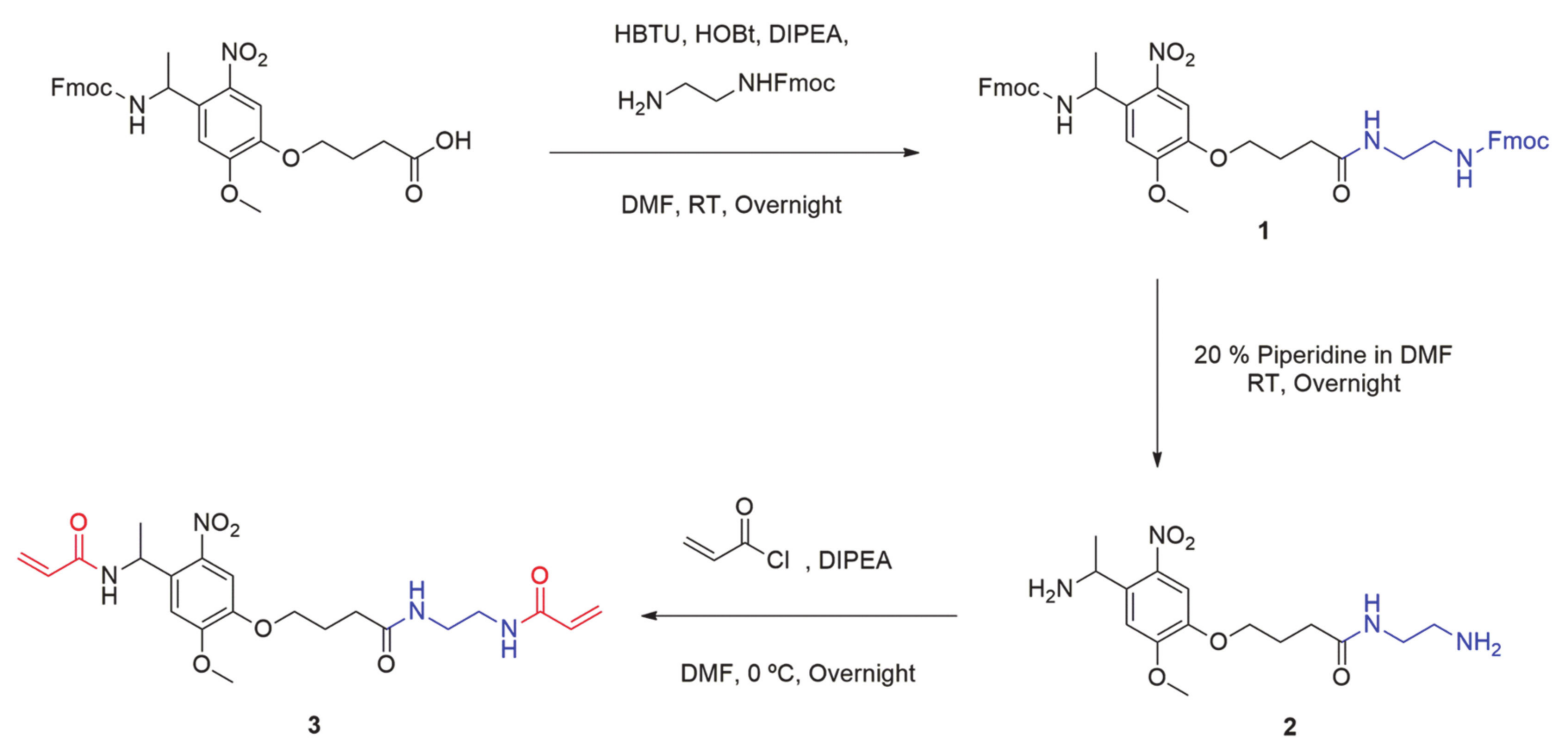
Synthetic pathway employed for the obtention of bisacrylamide photolinker, PL (product 3).

The commercially available Fmoc-photolinker carboxylic acid was employed as ONV source for the obtention of this crosslinker. The synthetic steps necessaries to obtain the molecule are summarized in Scheme 1. HBTU and HOBt benzotriazoles in basic conditions were employed to incorporate a nucleophilic amine group at the other end of the molecule. In this way, the correspondent activated ester for the amide formation was obtained by coupling the carboxylic end of the photolinker molecule and amine from mono-Fmoc diamine compound. The resulting double-Fmoc protected amide (product **1**) was analyzed by ^1^H NMR (Supplementary Materials, Figure S1) and Mass Spectrometry (Supplementary Materials, Figure S2). Then, both amine ends were deprotected by removing Fmoc groups in basic conditions employing a large excess of a solution of Piperidine/DMF at a 2:8 ratio, and the resulting mixture was evaporated under vacuum to obtain a crude containing the diamine-Photolinker (product 2). Due to the high polar and basic properties of this compound, the purification was easily carried out using a strong cation exchange retention technique with a solid phase extraction (SPE) SCX-2 cartridge. As the crude containing the diamine is passed through the Si-propylsulfonic acid (SCX-2) resin, the amine is retained by the strong cation exchange (SCX) column. By washing the column with MeOH in mild acidic conditions, non-basic impurities are not retained and are further removed. The product was subsequently released from the cartridge by elution with a 2 M solution of Ammonia in MeOH and solvent was dried, filtered, and evaporated. The obtained product 2 was analyzed by ^1^H NMR (Supplementary Materials, Figure S3).

Finally, product 2 was dissolved in anhydrous DMF under Nitrogen atmosphere, and DIPEA was added to keep the basicity of amine groups. Significant excess of acryloyl chloride was slowly added dropwise at 0 °C to this solution and kept overnight. Then, crude was evaporated under vacuum and dissolved in DCM. Then, the organic layer was extracted with a 5% aqueous solution of LiCl (to remove the remained DMF), brine and water, and evaporated under vacuum. The bisacrylamide photolinker (PL) product 3 was obtained as a pale-yellow solid. This novel compound was fully characterized by ^1^H NMR (Supplementary Materials, Figure S4), Mass spectrometry (MS) (Supplementary Materials, Figure S5), FTIR (Supplementary Materials, Figure S6), ^13^C NMR (Supplementary Materials, Figure S7), COSY 2D-NMR (Supplementary Materials, Figure S8), HMQC 2D-NMR (Supplementary Materials, Figure S9).

### 3.2. Photo-Lability Evaluation of PL

**Figure 1:**
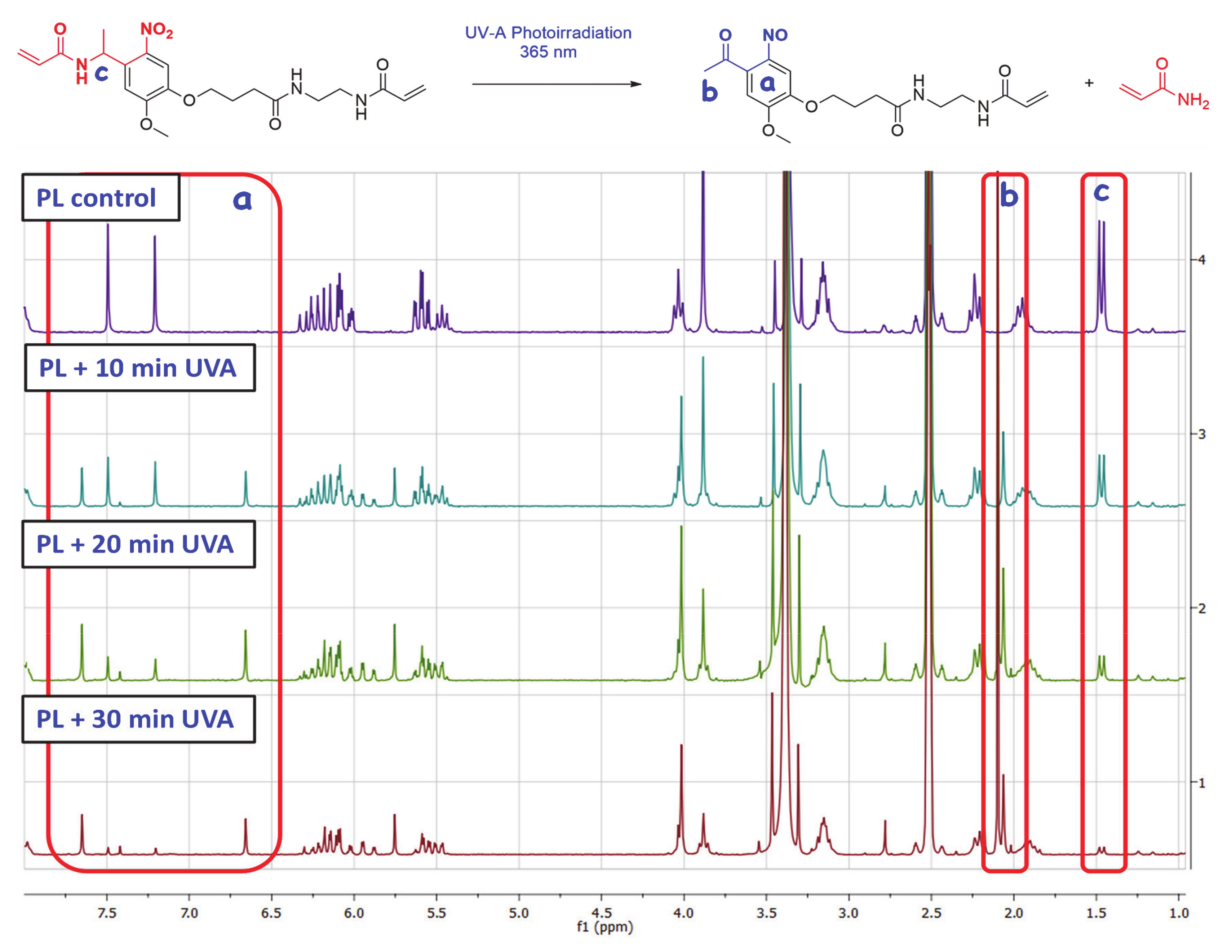
Upper image: Elimination reaction involved in the photodegradation of the synthetized photolinker (PL). Bottom image: ^1^H NMR comparative spectra for the three consecutive 10-min cycles of UVA irradiation.

UVA irradiation (365 nm) of the molecule containing the ONV photolabile moiety produces an intramolecular rearrangement through a complex three-step mechanism [35] (Figure 1-Upper image). This intramolecular photo-redox reaction of the nitroaromatic compound involves the overall reduction of the nitro group to nitroso, in concomitance with the oxidation of the heteroatom present in the ortho-benzyl position. Initiation is produced by the attacking nitro-group oxygen, which rapidly leads to the elimination of acrylamide and formation of the correspondent nitroso-benzaldehyde derivate. The incorporation of a methyl group on the benzyl position has been proved that enhances the cleavage kinetics by a 20-fold rate [36].

To confirm the UVA-sensitivity of synthetized product 3, it was diluted in DMSO-d_6_ (concentration of 2.5 mg/mL) and was transferred to a 1×1 cm quartz translucent cuvette. Then, the solution was exposed to three cycles of UVA irradiation (10 min of exposure each) using an irradiation wavelength of 365 nm at a distance of ~20 cm from the lamp focus. The energy registered at this point was 22.4 mW/cm^2^. After the three irradiation exposure times [Initial (PL + 10 min UVA) 10 min of irradiation (energy of 13.2 J/cm^2^), second 10 min of irradiation (PL + 20 min UVA) (accumulated energy of 26.4 J/cm^2^) and final 10 min of irradiation (PL + 30 min UVA) (accumulated energy of 39.6 J/cm^2^)], the solution was analyzed by ^1^H NMR, as can be observed in Figure 1-Bottom image. The same procedure was performed with an aluminum covered cuvette as a blank, with no variation in product ^1^H NMR spectrum after the three UVA exposure cycles (PL control).

Regarding initial (PL + 10 min UVA) and final NMR spectra (PL + 30 min UVA) of the compound, it is observable the apparition and progressive area increase of two peaks at ~6.66 ppm and ~7.65 ppm in the aromatic chemical shifts area (Figure 1, area a), due to the influence of ketone and NO electron withdrawing groups present in the aromatic ring of the degraded photolinker (dPL). It is also remarkable that a new peak at ~2.06 ppm (Figure 1, area b) corresponding with the methyl group of newly formed aryl ketone. In parallel, it is also observable the disappearance of the doublet peak at ~1.46 ppm (Figure 1, area c) corresponding to the α-methyl group of PL, confirming the elimination of acrylamide moiety to yield the ketone. The NMR calculated degradation ratio by the comparison of peak area from both compounds PL and dPL showed that the “degradation” yield for every irradiation pulse was ~45% for first 10 min, 72% for 20 min and 87% for 30 min (See Supplementary Materials, Figure S10).

This degradation was also confirmed by MS analysis (See Supplementary Materials, Figure S11), obtaining a m/z of 482.9 for the pre-irradiation sample (PL) and a m/z of 411.8 corresponding to PL after irradiation (dPL). A decrease of 71 units in the mass of the irradiated product was consistent with the mass of acrylamide sub product, which was generated in the degradation.

### 3.3. Synthesis of UVA-Sensitive Collagenase Nanocapsules (nCol-PL)

As we have introduced, this methodology is based on the method reported in previous works for the construction of a polymeric shell around collagenase enzyme [18,20,21]. In this method, nanocapsules were formed by in situ free-radicals polymerization of acrylamide-type monomers which were adsorbed on the macromolecule surface [37]. This interaction is favored by electrostatic interactions between monomers and collagenase surface, mainly hydrogen bonds. However, the relationship between electrostatics, protein interaction, and dynamic adsorption is an important one, but questions remain unanswered related to the physicochemical determinants of charge interactions. Moreover, these favourable interactions also positively contribute to the formation of the polymeric coating trough of the free-radicals polymerization reaction. The existing electron-rich hydroxyl and amino groups in the protein structure (i.e., in form of hydroxyproline, serine or lysine) can react with the free radicals formed during the decomposition of APS. These newly formed hydroxyl and amino radicals react, in turn, with the monomers that surround the protein, leading to the formation of the polymeric coating by the generation of covalent bonds.

**Figure 2:**
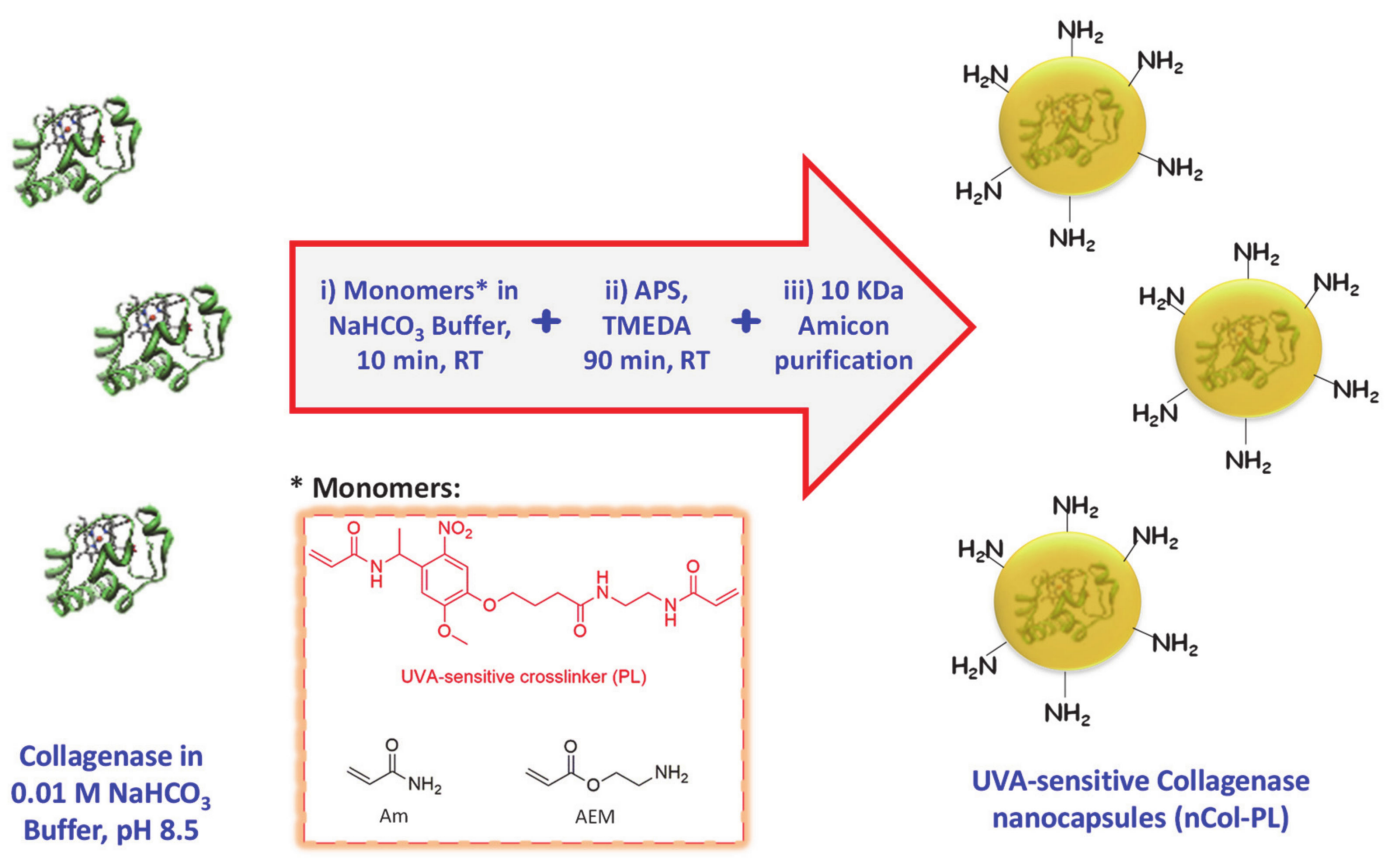
Schematic procedure for UVA-sensitive Collagenase nanocapsules synthesis. Asterisk indicates the monomers employed in the synthetic step i).

**Figure 3:**
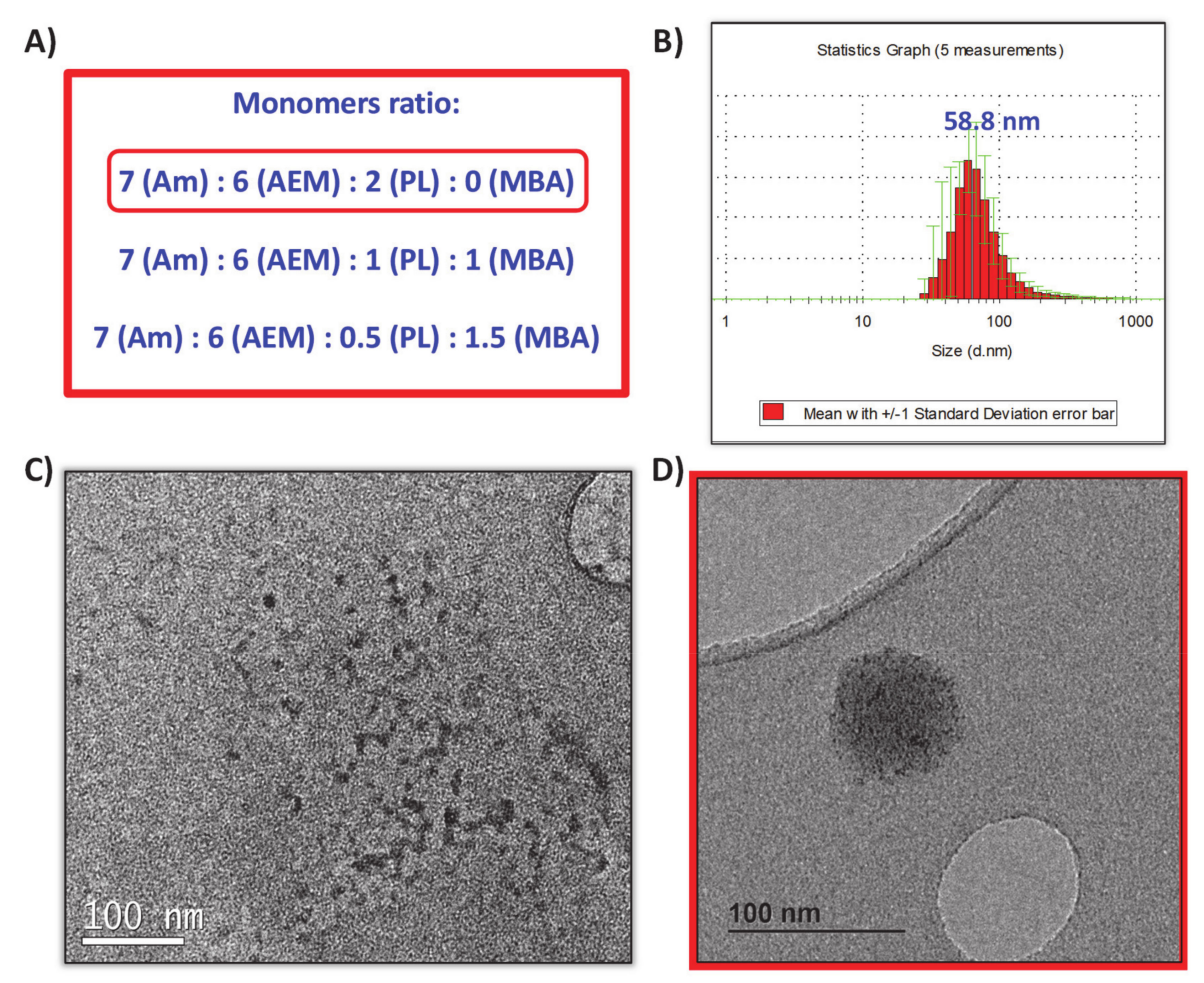
(**A**) Monomers ratio tested for the nanocapsules synthesis. (**B**) DLS obtained for nanocapsules synthetized with the 7:6:2 ratio. (**C**) TEM micrography of free collagenase before encapsulation. (**D**) TEM micrography of obtained nanocapsules.

Collagenase nanocapsules were synthesized using the free-radicals polymerization method, previously reported in our research works [18,19,20]. The incorporation of PL **(3)** as a crosslinker provide the capacity to respond to UVA radiation exposition. In this method, the monomers and proteins were mixed in a molar ratio of 2025:1 (monomers to protein) in aqueous solution at pH = 8.5 and then, after a short period of time required for the adsorption of the monomers on the protein surface, a radical initiator was added for inducing the formation of the protein capsule around the protein (Figure 2). Each monomer plays different role in the capsule formation. Am is a structural monomer, AEM provides colloidal stability to the capsule in physiological conditions, and PL is the monomer that provides the light-responsive capacity. A complete set of different nanocapsules were synthesized varying the ratio between monomers to produce capsules with variable degradability in the presence of UVA light. For this, a combination of both photolabile PL and non-degradable crosslinker N,N’-Methylenebisacrylamide (MBA) was employed (Figure 3A). Good results were achieved for the monomers ratio of 7 (Am):6 (AEM):2 (PL), thus discarding the employ of MBA as monomer.

Once the free-radicals reaction was finished, nanocapsules (nCol-PL) were purified by centrifugation in 10 kDa Amicon and they were preserved from light at 4 °C. The DLS analysis showed an average hydrodynamic size of ∼58 nm, as can be observed in Figure 3B. Z potential revealed a value of −11.0 mV, which was consistent with the presence of free amine groups in the polymeric framework of nanocapsules. Free collagenase and nCol-PL were also analyzed by TEM, showing that free collagenase (Figure 3C) had a ∼10 nm size, and then, when nanocapsules were synthetized (Figure 3D) they presented a size of ∼60 nm, consistent with DLS analysis. This suggest that nanocapsules are certainly “polymeric clusters” formed by the encapsulation of several collagenase enzymes.

### 3.4. In Vitro Cleavage Evaluation of nCol-PL and Enzymatic Activity Measurement

**Figure 4:**
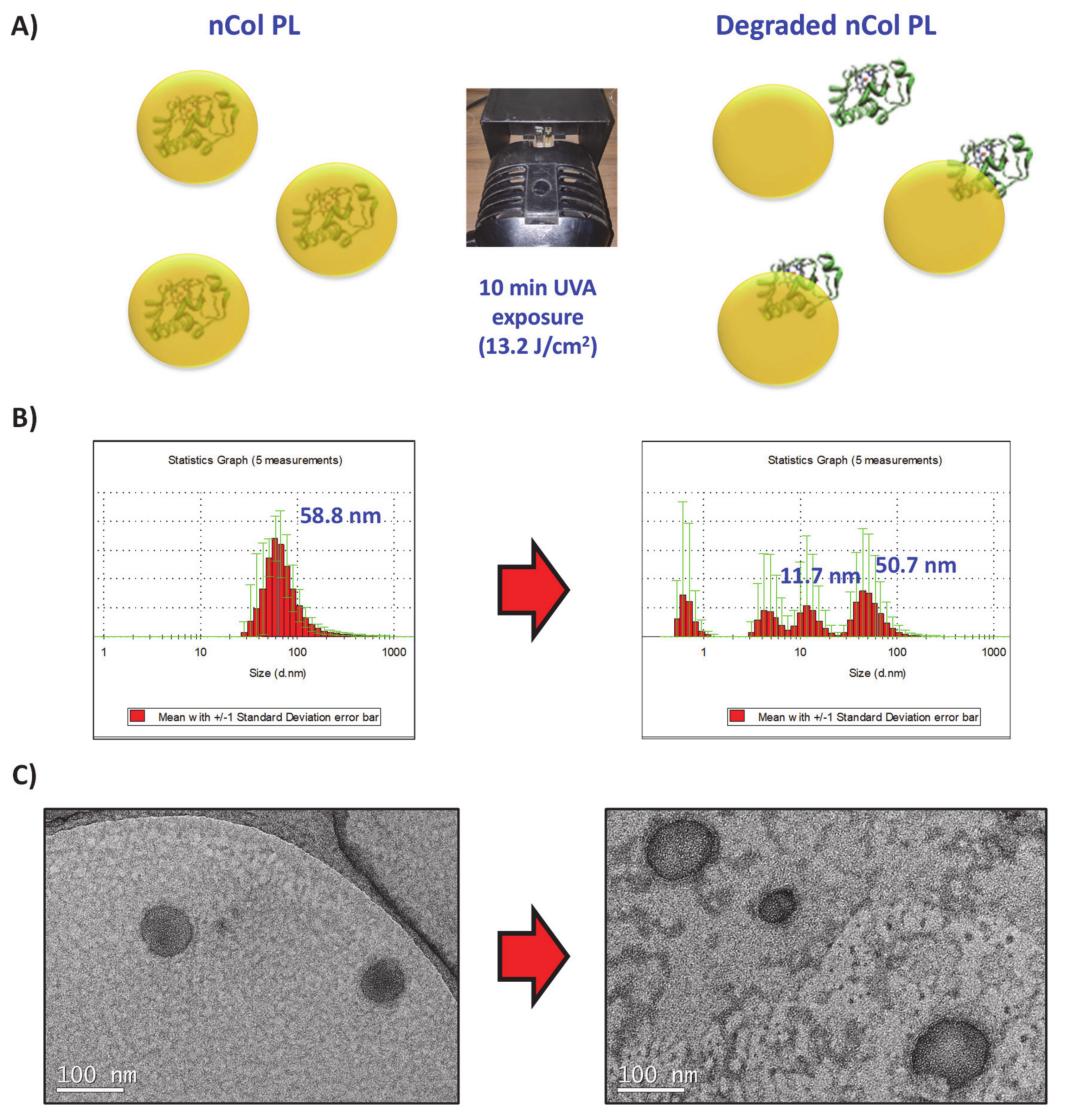
(**A**) UVA-sensitive nanocapsules exposed 10 min to UVA irradiation. (**B**) DLS analysis showed initial 58 nm size of nanocapusules (Left side), which after irradiation showed several size distributions that correspond with collagenase nanocapsules and free collagenase (Right side). (**C**) TEM micrographs also signaled the breakage of the nanocapsule framework.

Firstly, the UVA-sensitive behaviour of the produced nCol-PL was evaluated in an acellular aqueous media exposing the nanocapsules to UVA light. For this, a solution of nanocapsules with a protein concentration (calculated according to the protocol described in Section 2.3) of 50 µg/mL (corresponds to a theoretical collagenase activity of 14.5 U/mL) was introduced in a 1 × 1 cm quartz translucent cuvette and it was exposed to 10 min of UVA irradiation using an irradiation lamp with a wavelength of 365 nm at a distance of ∼20 cm from the lamp focus to the cuvette. Then, their size variation was evaluated by DLS and TEM. A summary scheme is shown in Figure 4A. The change in the hydrodynamic size of nanocapsules was observable from an initial size of ∼58 nm to a heterogeneous sizes distributions DLS diagram after 10 min of UVA exposure (Figure 4B). This result suggests that the crosslinker PL present in nCol-PL was degraded by UVA irradiation. TEM images showed that the polymeric framework that surrounds the enzyme was broken since free collagenase was also observable (Figure 4C). This breakage allowed the release of collagenase trapped within the nanocapsule, giving thus hydrodynamic sizes of an average ∼50 nm (related to non-degraded nCol-PL), ∼11 nm (corresponding to released collagenases) and smaller that correspond to polymeric slices derived from degraded capsules. These results indicated that 10 min UVA exposure was enough to degrade many of the nanocapsules present in the sample, but not the whole population of nanocapsules.

### 3.5. UVA-Degradation and Enzymatic Activity Evaluation Of Nanocapsules

After this preliminary evaluation, it was necessary to prove that the enzymatic activity remained after UVA irradiation, and the enzyme maintains its enzymatic properties. For this reason, the enzymatic activity of free collagenase was measured and nCol-PL following the protocol EnzChek Gelatinase/Collagenase Assay Kit. For this measurement were added 80 μL of 1× PBS, 20 μL of Collagen-FITC and 100 μL of each sample (Free collagenase as a control and both irradiated and not irradiated nCol-PL as samples) to a 96 well plate. All samples were standardized to an enzymatic concentration of 0.2 U/mL in 1× PBS solution at pH 7.4 at room temperature and their enzymatic activity was collected every 20 min for 2 h. The fluorescence intensity was measured in a fluorescence microplate reader (F_abs_ = 495 nm / F_em_ = 515 nm).

**Figure 5:**
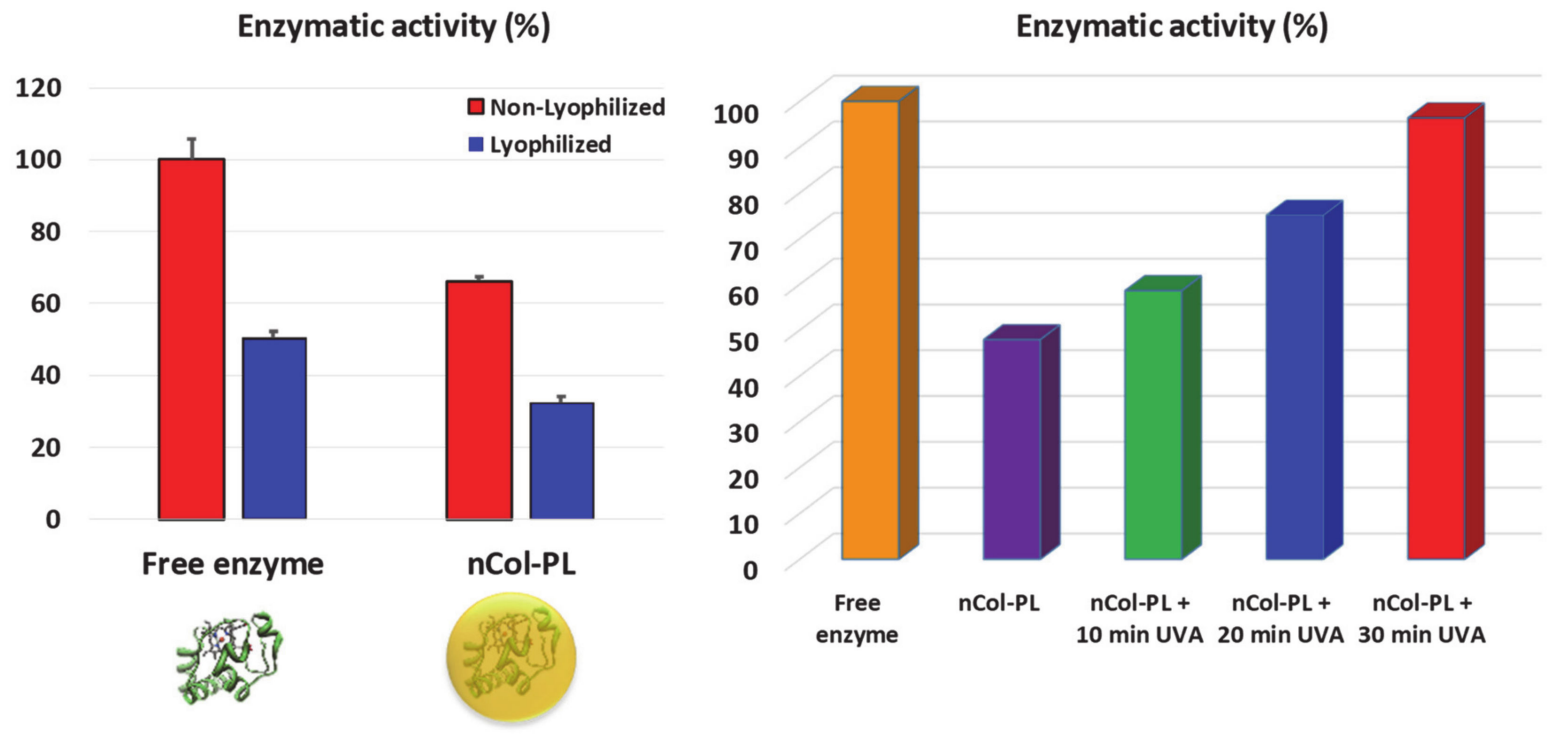
**Left:** Collagenase enzymatic activity (%) for free collagenase and nCol-PL nanocapsules measured before (red bars) and after (blue bars) lyophilization. **Right:** Collagenase activity (%) of free collagenase (orange bar), non-irradiated nCol-PL (purple bar) and irradiated nCol-PL with 10 min (green bar), 20 min (blue bar) or 30 min (red bar) of UVA light. Bars represent mean ± standard error of the mean (*n* = 3). *p* < 0.05 vs. free enzyme and nCol-PL. (Student’s two-tailed *t*-test).

In order to prove whether the nanocapsules could be stored in lyophilized form or in buffer solution, the enzymatic activity before and after lyophilization of free collagenase and nCol-PL was also tested. For this, 50 µg/mL of enzyme concentration for both samples were lyophilized and preserved −20 °C for a week. Then the enzymatic activity was measured as described above. As it is observable in Figure 5 in Left image, the enzymatic activity of free collagenase before lyophilization was significantly higher than nCol-PL due to the enzymes were encapsulated and therefore its enzymatic pocket was inaccessible for the substrate. After lyophilization, the enzymatic activity was newly measured, showing that the enzymatic activity for both free collagenase and nCol-PL decreased in a range of ∼50% comparing before lyophilization process. These results suggested that nanocapsules could not be stored by this method, thus, the decrease in the enzymatic activity was pronounced. Therefore, this method was discarded, and nCol-PL was thereby preserved in NaHCO_3_ buffer pH 8.5 at 4 °C.

nCol-PL UVA-sensitive degradation evaluation: nCol-PL degradation by UVA exposure was evaluated considering good clinical practices to determine suitable conditions to transfer this treatment to a future in vivo mouse model evaluation. According to previous studies [38], an exposure up to 30 J/cm^2^ UVA irradiation is below the Minimal Erytrema Dose (MED). This dosage has also been employed for in vivo experiments in mice [39]. In addition, considering that an accumulated energy of 39.6 J/cm^2^ has been found (in the Section 3.2) to be enough to degrade ~87% of a 2.5 mg/mL solution of PL, a 30 J/cm^2^ UVA irradiation was considered optimal to degrade the nCol-PL polymeric shell, and also to be accepted for clinical use. Thus, to evaluate the influence of UVA irradiation on enzymatic activity, the enzymatic activity of free collagenase (as a positive control) and nanocapsules was evaluated when UVA was irradiated in three pulses. As it is shown in Figure 5 at Right image, the enzymatic activity when the enzyme was encapsulated was a ~40–50% less than free collagenase. This decrease was due to the inability of the enzyme to degrade the substrate because it was covered by the polymeric framework of nanocapsule. Then, when nCol-PL were exposed to three pulses of 10 min UVA (10, 20 and 30 accumulated minutes, respectively), it is observable that the enzymatic activity of collagenase was recovered after each UVA exposure until reach a similar value to the original activity of free collagenase when 30 min of UVA were applied. This result is consistent with the fact that once the photolinker from polymeric framework of nanocapsules was degraded, the enzymes were liberated from polymeric coating, and thus, the enzymatic activity in the medium increases. This effect reinforces our previsions that nanocapsules acts as reservoir of enzyme and their degradation can be controlled and modulated with the time of UVA exposition.

### 3.6. Nanocapsules Kinetics for Collagen Degradation Within Collagen Gels

**Figure 6:**
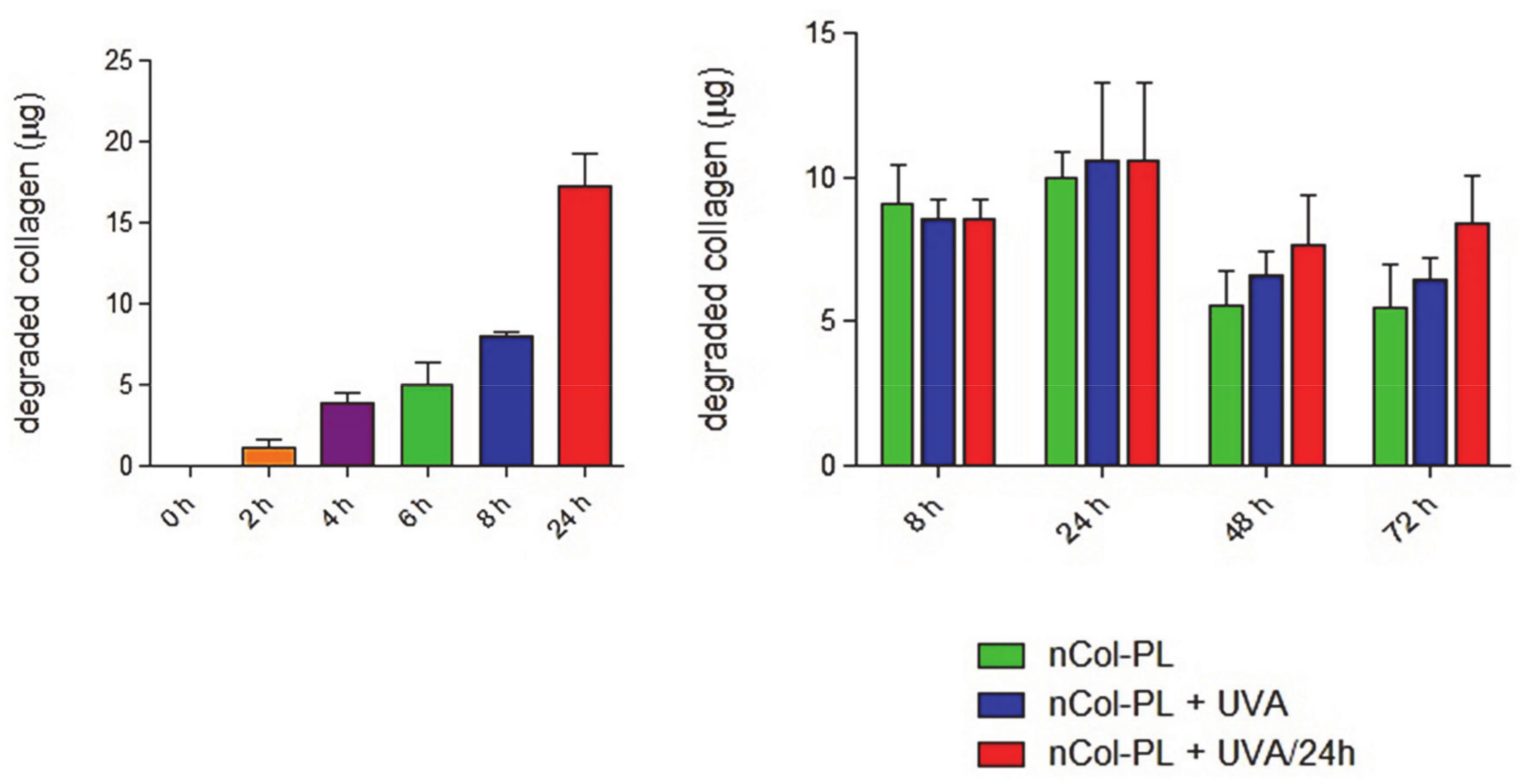
Collagenase kinetics of collagen degradation. **Left:** Quantification of the amount of degraded collagen (µg) throughout 24 h, for the indicated time-points. **Right:** Quantification of the amount of degraded collagen (µg) throughout 72 h for the indicated time-points, after incubation with non-UVA treated nanocapsules (nCol-PL, green bar), single-UVA treated nanocapsules (nCol-PL + UVA, blue bar) or nanocapsules treated with UVA pulse each 24 h (nCol-PL + UVA/24 h), red bar). Bars represent mean ± standard error of the mean (*n* = 3).

To study the collagenase kinetics of collagen degradation in a more realistic conditions of diseased skin, the enzymatic activity of UVA-activated nCol-PL along three days was also evaluated by employing as substrate a three-dimensional collagen matrix. For this, a synthetic collagen gel (1.46 mg/mL of collagen) was employed as substrate, which was incubated into a 96-well plate for an easy analysis. A low concentration of nCol-PL (20 µg/mL) was used to be able to quantify the progressive degradation of the gel over 24 h. These nanocapsules were added on top of the collagen gel-seeded wells, and the obtained samples were exposed to UVA radiation during 15 min (exposure energy of 30 J/cm^2^). Then, the supernatants corresponding to each time sample were carefully collected after 0 h, 2 h, 4 h, 6 h, 8 h and 24 h, respectively. A sample without nCol-PL was also prepared as a control for collagen degradation. To stop the enzymatic activity after the collection of the supernatants, they were quickly frozen at −80 °C. Then, the amount of soluble collagen was determined using the Soluble Collagen Quantification Assay Kit (Fluorometric assay). It is based on the quantification of collagen polypeptides derived from enzymatic digestion of the gel. For the identification of these peptides, they were labelled with a fluorescent probe, according with the protocol described in the assay kit, and then, the fluorescence intensity was measured at λ_ex_ = 375 nm/λ_em_ = 465 nm. As the kit quantify indirectly the degraded collagen by fluorescent labelling the collagen degradation products, we carried out the assay considering the wells with synthetized collagen gels without nCol-PL as control samples and supernatant from wells with gel digested with nCol-PL as samples, both for each incubation time. The obtained fluorescence was proportional to the amount of soluble collagen present in supernatant sample. The amount of accumulated degraded collagen (µg) obtained for each time of incubation (all data were corrected with untreated control) are shown in Figure 6, left. The results showed a progressive degradation of the collagen gel by the enzymatic activity of collagenase released from UVA-activated nCol-PL, giving that the collagen degraded by nanocapsules (20 µg/mL) was around 17 µg after 24 h. The mean collagen degradation capacity of these nanocapsules was 0.8 µg per hour. nCol-PL collagenase activity does not seem to decrease after 24 h of incubation.

In order to approach our experiments to a clinical guideline, the variation of the enzymatic activity during the time and effect of the application of three pulses of UVA (one per day) to nanocapsules were evaluated. For this, the amount of collagen degraded was measured during a period of 72 h, as can be observed in Figure 6, right. The same experiment was performed as described before, but in this case, the enzymatic activity of nCol-PL (20 µg/mL) was evaluated during 72 h by comparing the enzymatic activity obtained in three different cases. These conditions were nCol-PL without UVA exposure (green bar), with only one exposure to UVA (blue bar) and with three UVA exposures at 24, 48 and 72 h (red bar). Through this, it was possible to evaluate if the enzymatic activity increases when UVA was applied comparing with non-treated nanocapsules. The results showed that non-UVA treated nanocapsules possessed high enzymatic activity at 8 h and 24 h times, maybe due to non-encapsulated collagenase or premature breakage of nanocapsules. However, after 48 h and 72 h this enzymatic activity decreases noteworthy due to the high lability of non-encapsulated collagenase. However, when one exposure to UVA was applied to nCol-PL, the amount of collagen degraded after 24 h, 48 h and 72 h reached a higher value, compared with the non-treated sample. Interestingly, when three UVA exposure pulses were applied (one each 24 h of incubation), it was observed that each applied pulse increased the amount of degraded collagen. Compared with one UVA-pulse and non-irradiated samples, the difference in the amount of collagen was higher in correlation with the number of UVA pulses. This result suggests that the breakage and opening of nanocapsules is a slow process and collagenase was slowly released from polymeric network. This effect also reinforces our hypothesis that these nanocapsules could acts as a reservoir of collagenase that can be easily controlled and modulated with an easy-to focus stimulus, such as UVA irradiation, given that the collagen degradation after 24 h was higher in the case of UVA treated nanocapsules face to untreated ones. In this way, higher times of UVA exposures or some UVA exposure cycles could increase the release of collagenase from nanocapsules, depending on therapeutic or clinical dosage necessary in future applications.

### 3.7. Evaluation of nCol-PL Cytotoxicity

**Figure 7:**
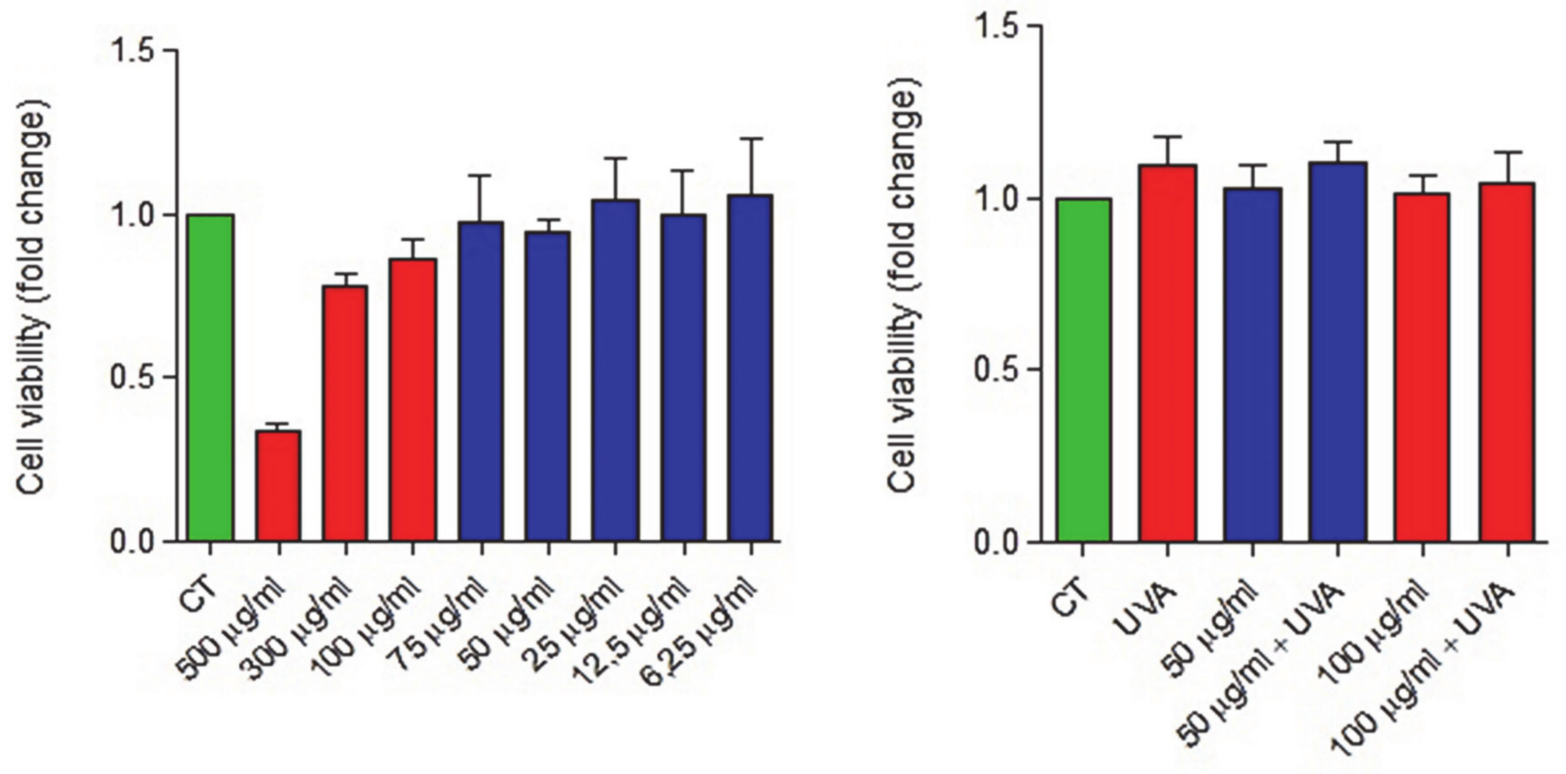
Biocompatibility evaluation of nCol-PL in primary skin fibroblasts cultures. **Left:** Cell viability (fold-change) of a primary skin fibroblasts culture after its treatment with increasing concentrations of nCol-PL. **Right**: Cell viability (fold-change) of a primary skin fibroblasts culture after its treatment with 100 µg/mL and 50 µg/mL nCol-PL in presence or absence of UVA irradiation. Bars represent mean ± standard error of the mean. The assays were performed at least twice, six replicates for each condition per assay.

After the evaluation of the modulable enzymatic activity, biocompatibility of these nanocapsules was tested in vitro in the presence of healthy patients’ skin fibroblasts. The effect on the viability of primary skin fibroblast cultures was evaluated for nCol-PL, but also for UVA irradiation. For this, cells were either untreated or treated with a wide range of increasing nCol-PL concentrations: 6.25 µg/mL, 12.5 µg/mL, 25 µg/mL, 50 µg/mL, 75 µg/mL, 100 µg/mL, 300 µg/mL and 500 µg/mL. Cell viability was measured by the quantification of absorbance at 450 nm 24 h after the treatment, following the WST-1 protocol. Results showed that cell viability was almost 100% in doses below 75 µg/mL (Figure 7, Left). Although data obtained for low doses did not show a clear dose-dependency, this effect can be attributed to the marked dispersion between samples. Nonetheless, we can state clearly that the administration of nCol-PL in low doses did not affect to the cell viability.

However, higher doses than 75 µg/mL started to affect the cell viability in a range of 10–20% until 300 µg/mL doses, while 500 µg/mL of nCol-PL significantly reduced cell viability to a value of 35%. This group had previously described similar nanocapsules suitable for skin fibrosis treatment [20]. In this work, the use of 300 µg/mL of collagenase nanocapsules lacked cytotoxicity in mice and was effective in the reduction of the collagen area. Thus, based on these results and to facilitate the comparison between the therapeutic potential of both types of collagen nanocapsules, dose of 300 µg/mL can be stablished as a biocompatibility maximum limit for their evaluation in clinical uses, given that the influence in viability at this concentration started to be significative.

Once we determined that the treatment with doses equal or below 300 µg/mL of nCol-PL lacked significant cytotoxicity, the possible effect on cell viability caused by the exposure of the cells to 30 J/cm^2^ UVA light (needed for nCol-PL degradation) was also assessed. In this case, an intermediate dosage of 100 µg/mL (limit dose which viability started to be affected) and 50 µg/mL (did not affect viability) nCol-PL concentrations were chosen for this assay. Cells were incubated with 50 µg/mL and 100 µg/mL of nCol-PL and both were subsequently treated or untreated with a single 30 J/cm^2^ UVA pulse to evaluate the influence of UVA-mediated degradation of nanocapsules on viability. The cells were also treated with a single 30 J/cm^2^ UVA pulse without addition of nCol-PL, in order to observe the endogenous effect of UVA radiation. The measurement of the absorbance at 450 nm 24 h, after treatment, showed that any of the tested conditions had a significative effect on the cell viability (Figure 7, Right). Therefore, the exposure to a 30 J/cm^2^ UVA light, described as suitable for human clinical use, can indeed be considered safe and optimal as therapeutic dose.

## 4. Conclusions

A new approach for treating fibrotic diseases in localized areas through the release of collagenase from within UVA-degradable polymeric nanocapsules has been presented. The novel compound Bisacrylamide-Photolinker has been synthetized and deeply characterized, with the aim of providing UVA-sensitivity properties to nanocapsules. These nanocapsules have been successfully prepared through free-radicals polymerization using acrylamide-type monomers. The employ of UVA-sensitive collagenase nanocapsules provides a triple advantage in comparison with the conventional administration of free enzymes. Firstly, the use of these capsules for transporting the proteolytic enzymes solves the problem associated with a lower stability of the enzymes in physiological conditions. Polymeric nanocapsule protect the enzymes against external factors, present in the diseased tissue, such as other proteolytic enzymes, oxidative agents and temperature increases. Secondly, the capacity to release the housed enzyme only if UVA radiation is applied allows a precise control of the administered dose along the time. Higher enzymatic activities were observed when additional UVA pulse were applied to nCol-PL. This fact avoids the apparition of side effects, which usually accompany the use of proteolytic enzymes, provoked by an excessive tissue degradation. Therefore, it would maximize the efficacy of the therapy in a focused area. Finally, the choice of UVA radiation for triggering the release of collagenase might provide additional benefits for the therapy, due to its potential anti-inflammatory effects and its capacity to inhibit the collagen synthesis in the irradiated area of the diseased tissue.

The administration of nanocapsules can be carried out locally, providing greater advantages for the treatment of localized lesions caused by fibrosis. Their systemic use (i.e., intravenously) followed by local activation in patients with other fibrotic diseases, such as localized or systemic scleroderma, is a potential application to be explored. In all cases, the fact that the collagenase release takes place only if UVA is applied, will focus the therapeutic effect exclusively in the diseased zones, thereby reducing the apparition of side effects in healthy tissues. A significant therapeutic improvement is expected, as a result of the the synergy between these capacities and the easily-controlled release of the proteolytic enzyme.

## Data Availability

Not applicable.

## Supplementary Materials

The following are available online at https://www.mdpi.com/article/10.3390/pharmaceutics13040499/s1, Figure S1: 1H NMR spectrum of Fmoc-PL-NHFmoc (product 1), Figure S2: Maldi-TOF/TOF MS analysis of Fmoc-PL-NHFmoc (product 1), Figure S3: 1H NMR of Diamine-Photolinker (product 2), Figure S4: 1H NMR spectrum of Bisacrylamide-Photolinker (product 3), Figure S5: ESI-MS analysis of Bisacrylamide-Photolinker (product 3), Figure S6: FTIR spectrum of Bisacrylamide-Photolinker (product 3), Figure S7: 13C-NMR spectrum of Bisacrylamide-Photolinker (product 3), Figure S8: COSY 2D-NMR spectrum of Bisacrylamide-Photolinker (product 3), Figure S9: HMQC 2D-NMR spectrum of Bisacrylamide-Photolinker (product 3), Figure S10: 1H-NMR spectra for Control PL and for PL irradiated with one pulse of UVA for 10 minutes (PL+10 min UVA), two pulses of 10 + 10 min (PL+20 min UVA), and three pulses of 10 min each (PL+30 min UVA), Figure S11: ESI-MS analysis of Bisacrylamide-Photolinker before (PL) and after UVA irradiation (dPL). In brief, ^1^H-NMR, MS, FTIR, ^13^C-NMR, COSY 2D-NMR and HMQC 2D-NMR analyses of different synthetized molecules are included in Supplementary data. A detailed ^1^H-NMR and MS analysis of PL after UVA treatment is additionally included.
