# Supplementary material for: UVA-Degradable Collagenase Nanocapsules as a Potential Treatment for Fibrotic Diseases
Source: Pharmaceutics. 2021 Apr 6;13(4):499. doi: 10.3390/pharmaceutics13040499 (PMC8067494; doi:10.3390/pharmaceutics13040499)
Supplement: Supplementary file 1 [file pharmaceutics-13-00499-s001.pdf]

pharmaceutics

Supplementary Materials: UVA-Degradable Collagenase Nanocapsules as a Potential Treatment for Fibrotic Diseases

Víctor M. Moreno, Carolina Meroño, Alejandro Baeza, Alicia Usategui, Pablo L. Ortiz-Romero, José L. Pablos and María Vallet-Regí

Table of Contents

Characterization Of Compounds

Table of Contents

1. Double-Fmoc Protected Amide (Product 1)
2. Diamine-Photolinker (Product 2)
3. Bisacrylamide Photolinker, PL (Product 3)
4. UVA Degradation of PL to dPL

1. Double-Fmoc Protected Amide (Product 1)

^{1}H NMR spectrum

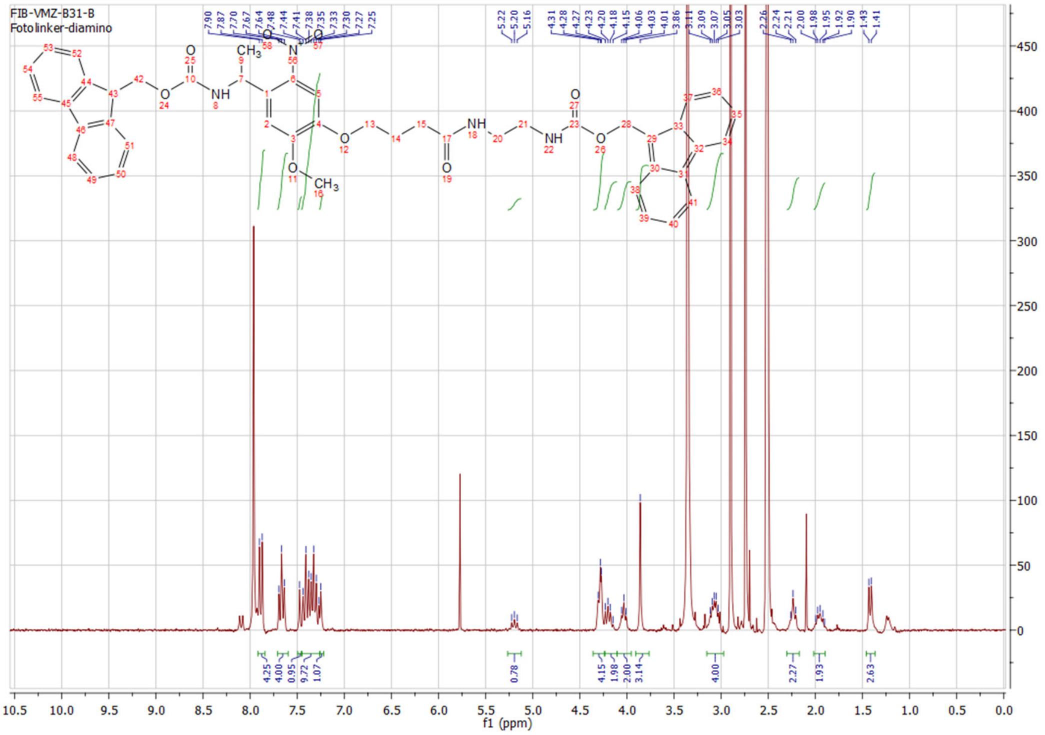

Figure S1. ^{1}H NMR (250 MHz, MeOD) spectrum of Fmoc-PL-NHFmoc **(1).** Solvent residual peaks observed for DCM (5.76 ppm), Acetone (2.09 ppm) and DMF (7.95 ppm, 2.89 ppm, 2.73 ppm).

Maldi-TOF/TOF spectrum

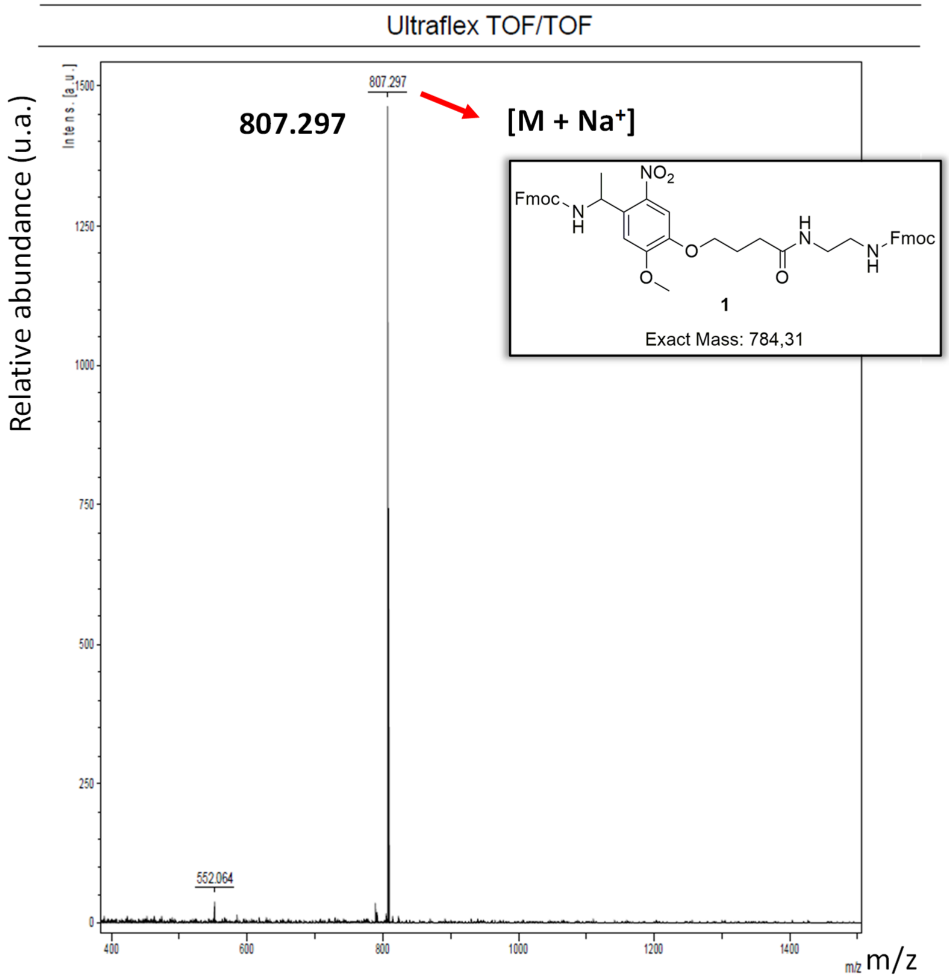

Figure S2. Matrix-Assisted Laser Desorption/Ionization-Time-Of-Flight (Maldi-TOF/TOF) MS analysis of Fmoc-PL-NHFmoc **(1).** Chemical Formula: $C_{45}H_{44}N_4O_9$. Exact Mass (m/z): 784,31.

2. Diamine-Photolinker (Product 2)

^{1}H NMR spectrum

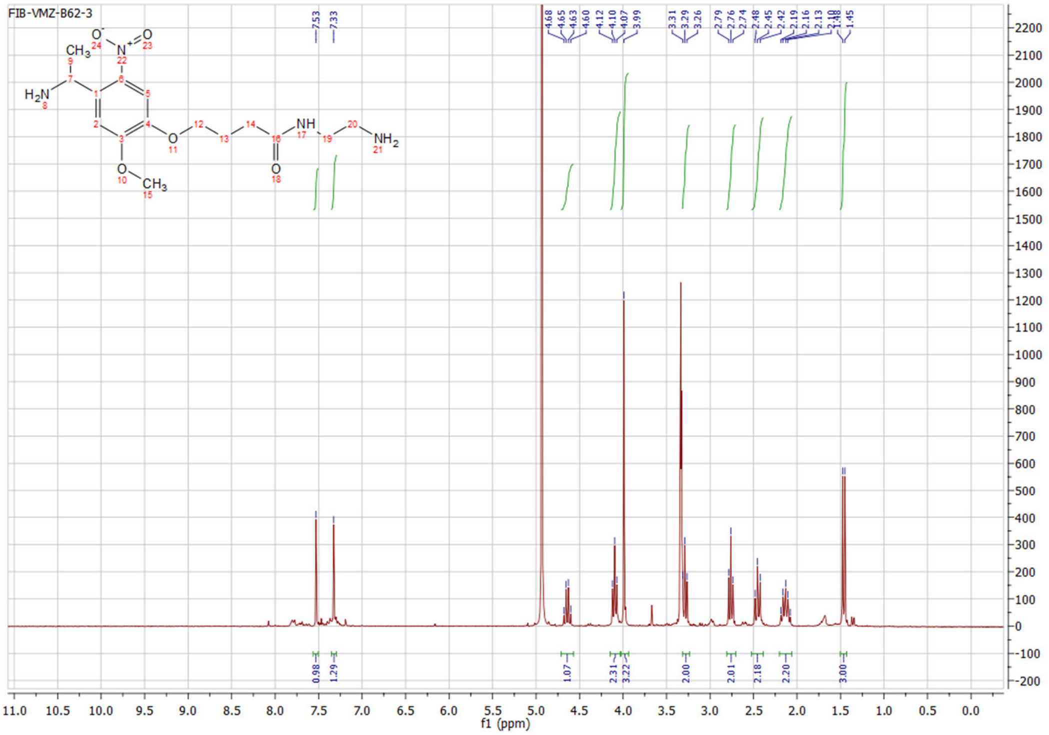

Figure S3. ^{1}H NMR (250 MHz, MeOD) spectrum of Diamine-Photolinker **(2).**

3. Bisacrylamide Photolinker, PL (Product 3)

^{1}H NMR spectrum

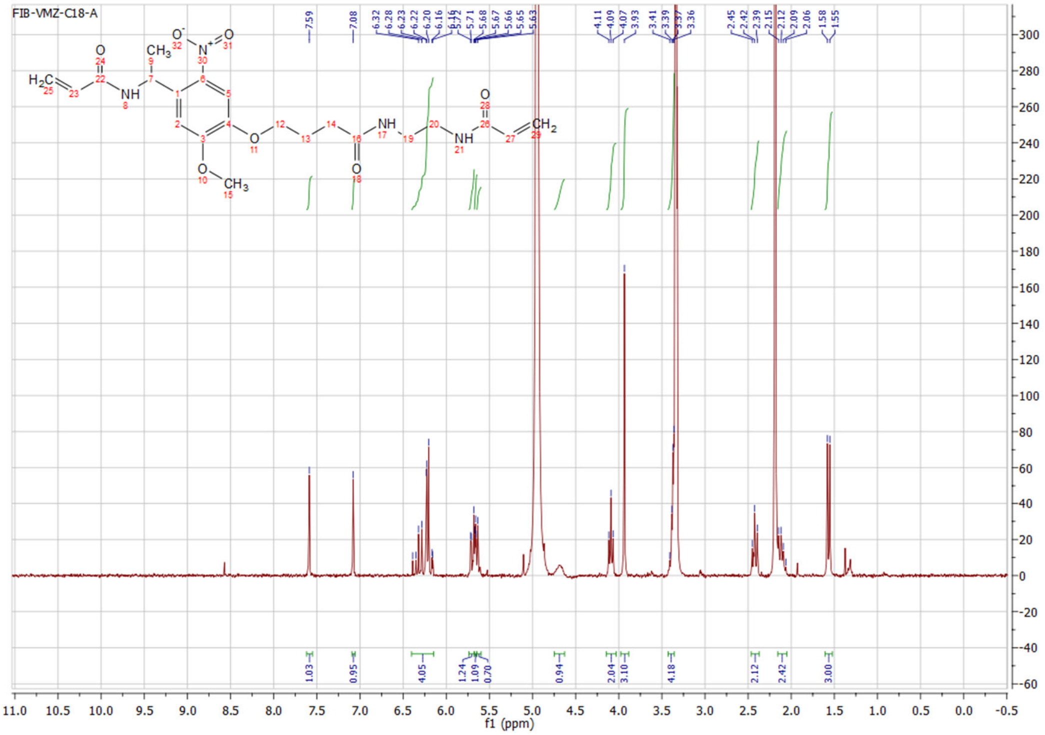

Figure S4. ^{1}H NMR (250 MHz, MeOD) spectrum of Bisacrylamide-Photolinker **(3).**

MS spectrum

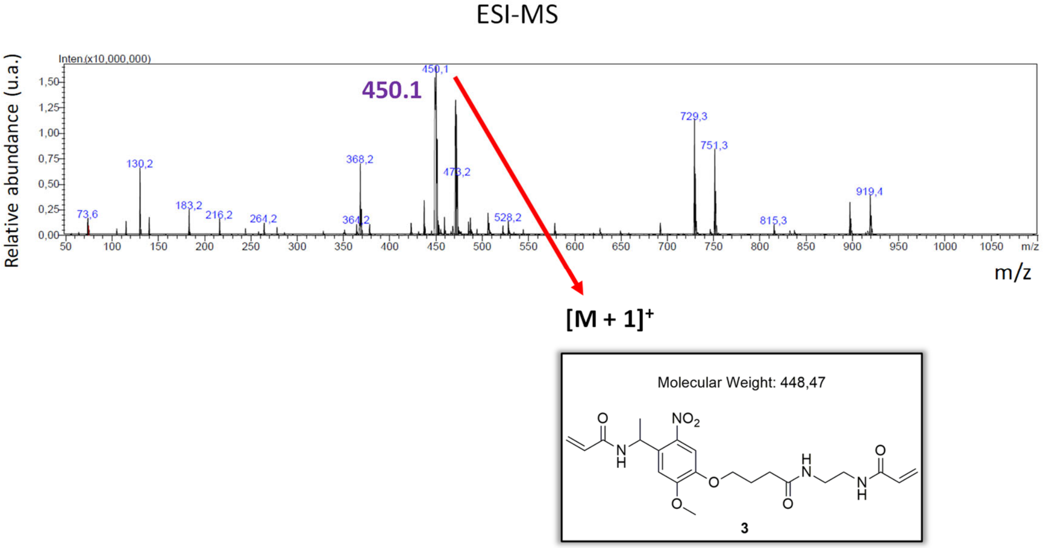

Figure S5. Electrospray Ionization (ESI-MS) MS analysis of Bisacrylamide-Photolinker **(3)**. Chemical Formula: $C_{21}H_{28}N_4O_7$. Exact Mass (m/z): 448,47.

FTIR spectrum

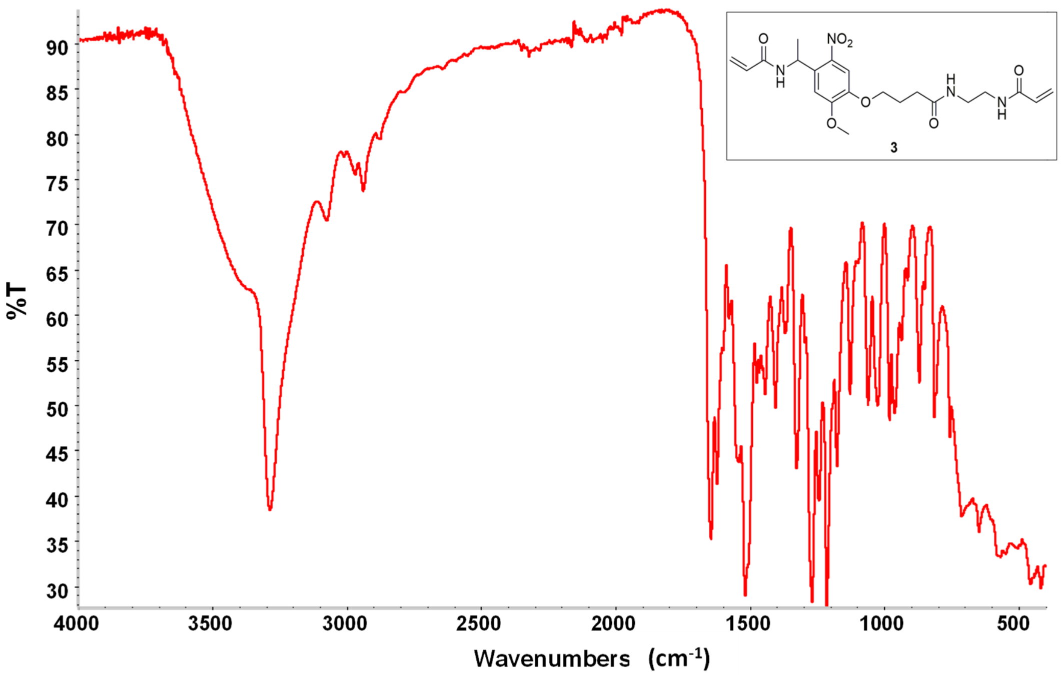

Figure S6. FTIR spectrum of Bisacrylamide-Photolinker **(3)**. Intense peaks for C-O stretching at ~1210 cm^{-1} and ~1280 cm^{-1} were assigned to aryl alkyl ethers. Peaks for N=O stretching at ~1550 cm^{-1} (asymmetric) and ~1330 cm^{-1} (symmetric) were attributed to aromatic nitro group. Intense peak for aromatic C=C stretching at ~1500–1520 cm^{-1} were attributed to aromatic ring. Intense peak for C=C stretching at ~1630 cm^{-1} corresponded to vinyl groups. Strong C=O peak at ~1650 cm^{-1} relative to C=O from amides. C-H stretching peaks at ~2800–3100 cm^{-1} related to vinyl groups and aromatic ring. Strong N-H stretching peak at 3300 cm^{-1} related to amides. Broad O-H stretching band at ~3300–3700 cm^{-1} was assigned to water traces present in the sample.

^{13}C NMR spectrum

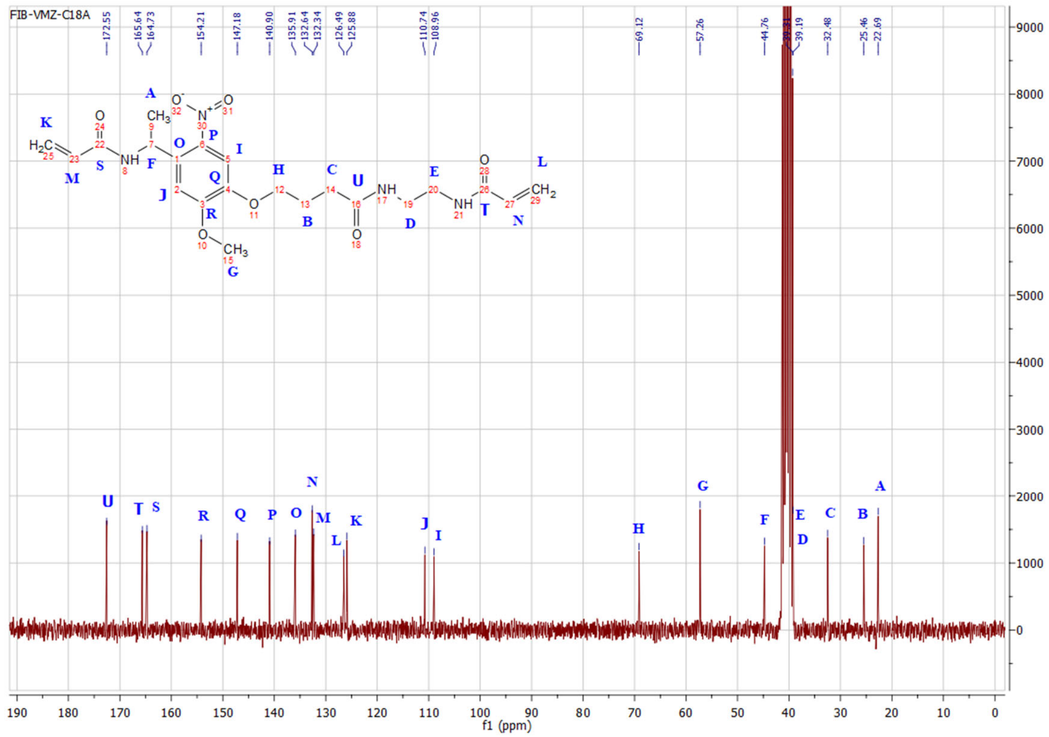

Figure S7. ^{13}C-NMR spectrum of Bisacrylamide-Photolinker **(3)**. ^{13}C-NMR (63 MHz, DMSO) δ 172.55, 165.64, 164.73, 154.21, 147.18, 140.90, 135.91, 132.64, 132.34, 126.49, 125.88, 110.74, 108.96, 69.12, 57.26, 44.76, 39.31, 39.19, 32.48, 25.46, 22.69.

COSY 2D-NMR spectrum

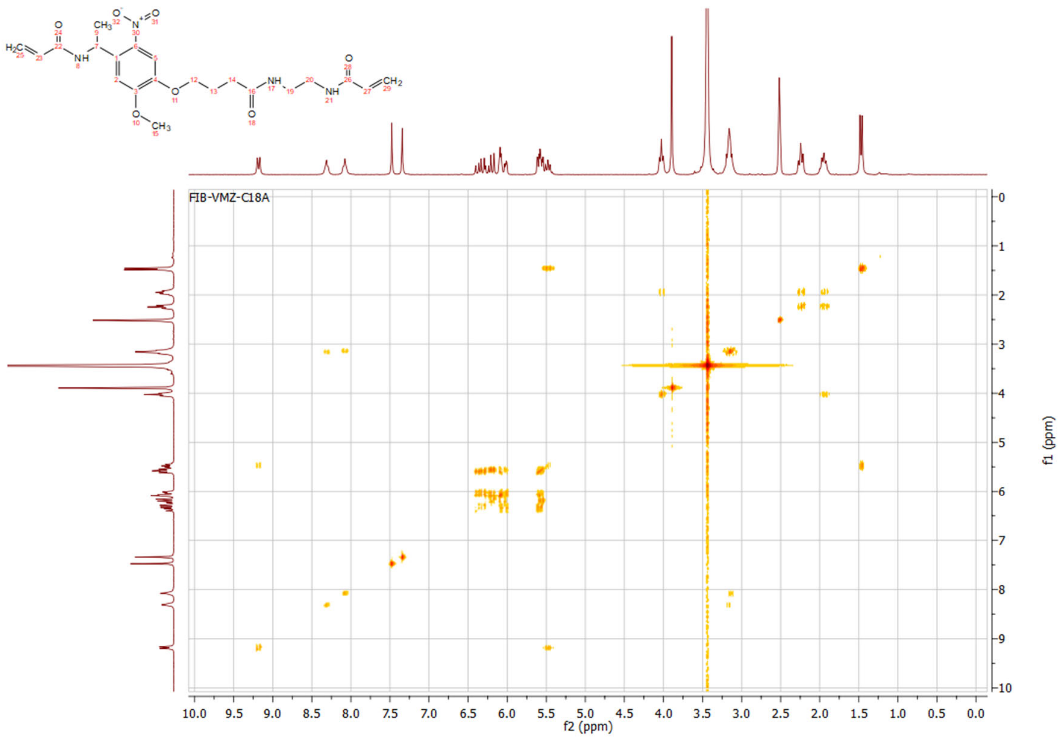

Figure S8. COSY 2D-NMR spectrum of Bisacrylamide-Photolinker **(3)**.

HMQC 2D-NMR spectrum

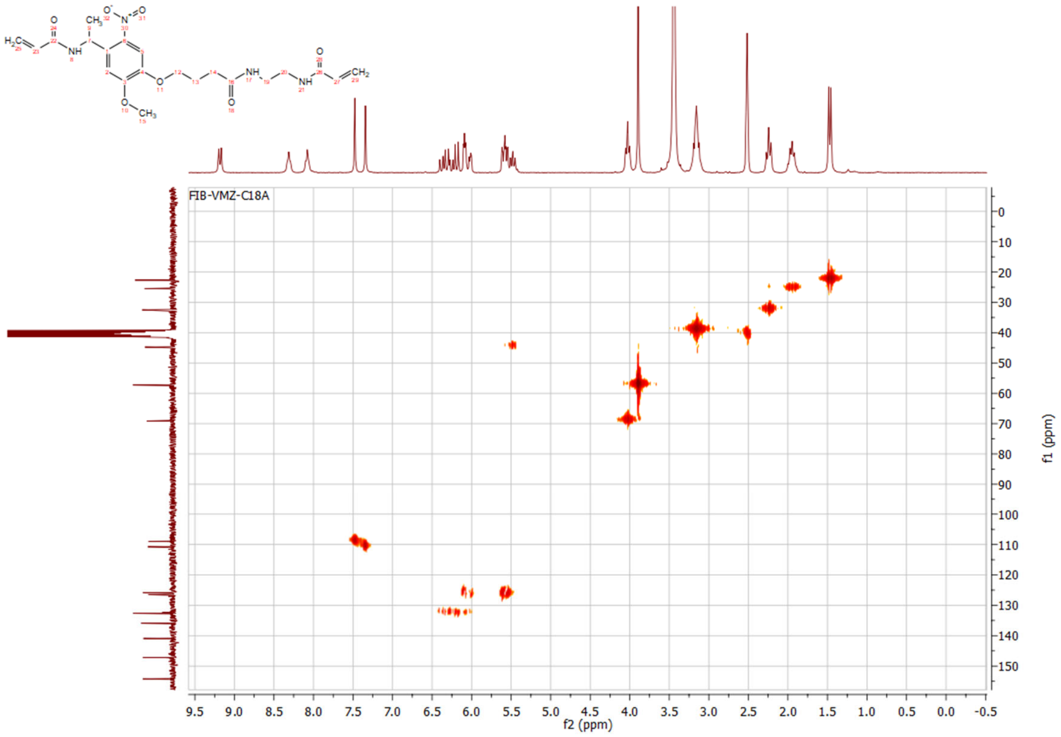

Figure S9. HMQC 2D-NMR spectrum of Bisacrylamide-Photolinker **(3)**.

4. UVA-Induced Degradation of PL to dPL

<u>^{1}H-NMR calculated yield:</u> The integral area of a ^{1}H peak corresponding to Photolinker (PL) and a ^{1}H peak corresponding to degraded Photolinker (dPL) was calculated after each irradiation pulse of 10 min of UVA. They were compared with the ^{1}H signal of an internal standard (considered integral of CH_2 at 1.95 ppm). Comparing the equivalents obtained from integral area of both products, it was calculated the molar ratio between both compounds. The molar ratio for each compound was obtained as: % = (^{1}H area of compound X / total ^{1}H area of PL and dPL) × 100.

NMR for UVA-induced degradation of PL to dPL

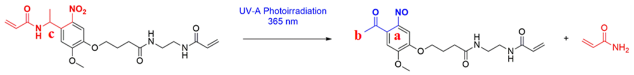

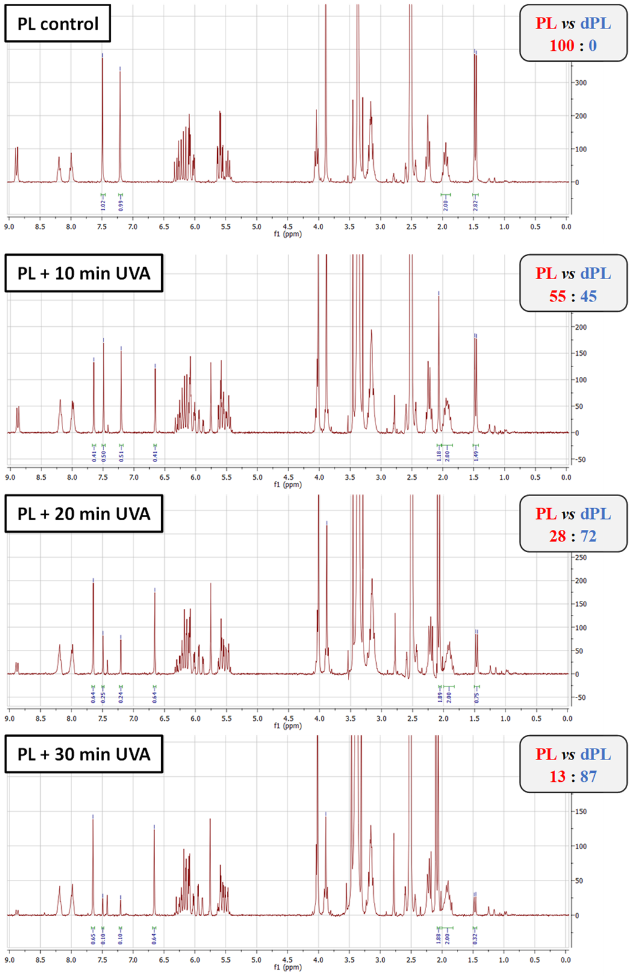

Figure S10. ^{1}H-NMR spectra for Control PL and for PL irradiated with one pulse of UVA for 10 minutes (PL+10 min UVA), two pulses of 10 + 10 min (PL+20 min UVA), and three pulses of 10 min each (PL + 30 min UVA). Calculated ratio between PL and dPL for each sample is represented in the right boxes.

MS for PL and dPL compounds

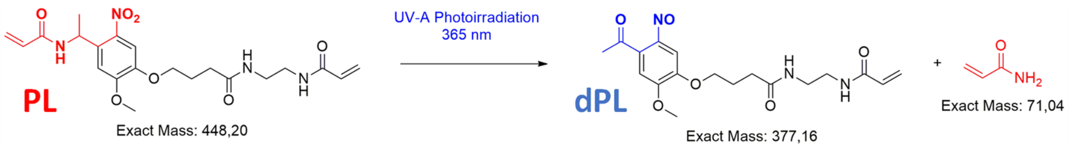

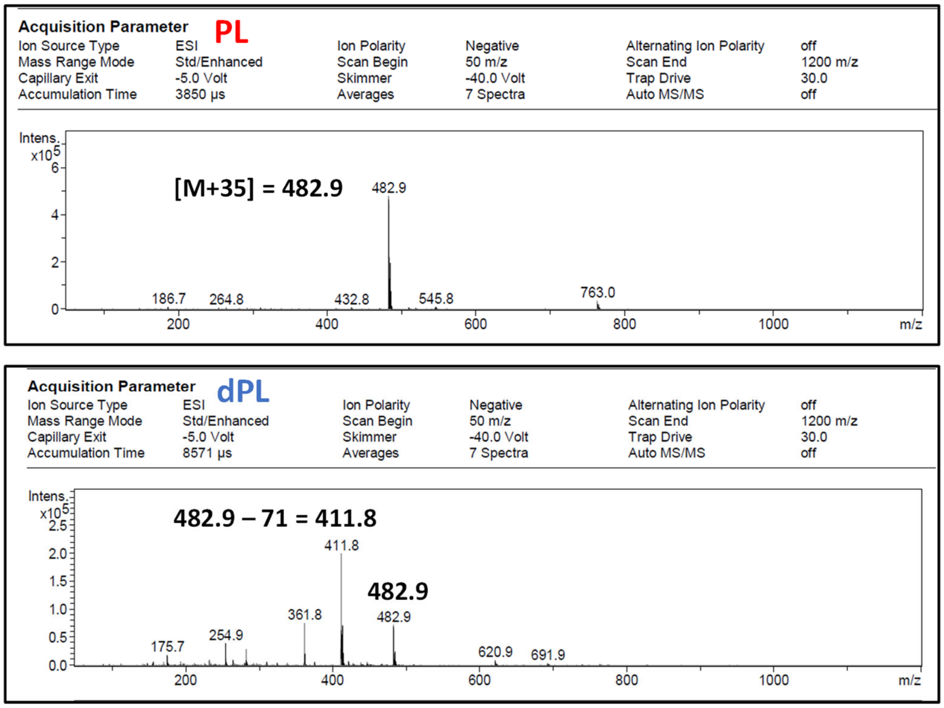

Figure S11. Electrospray Ionization MS (ESI-MS) analysis of Photolinker (PL) before (PL) and after UVA irradiation (dPL). m/z of 482.9 correspond to PL. m/z of 411.8 correspond to degraded PL (dPL). This is consistent with the PL mass–71 (mass of acrylamide degradation subproduct).
